# Multiple Myeloma Cells Alter Adipogenesis, Increase Senescence-Related and Inflammatory Gene Transcript Expression, and Alter Metabolism in Preadipocytes

**DOI:** 10.3389/fonc.2020.584683

**Published:** 2021-02-18

**Authors:** Heather Fairfield, Samantha Costa, Carolyne Falank, Mariah Farrell, Connor S. Murphy, Anastasia D’Amico, Heather Driscoll, Michaela R. Reagan

**Affiliations:** ^1^ Center for Molecular Medicine, Maine Medical Center Research Institute, Scarborough, ME, United States; ^2^ School of Medicine, Tufts University, Boston, MA, United States; ^3^ Graduate School of Biomedical Science and Engineering, University of Maine, Orono, ME, United States; ^4^ Biology Department, University of Southern Maine, Portland, ME, United States; ^5^ Biology Department, Norwich University, Northfield, VT, United States

**Keywords:** myeloma, bone marrow, adipocytes, senescence, microarray, mesenchymal stromal cells (MSCs), preadipocytes

## Abstract

Within the bone marrow microenvironment, mesenchymal stromal cells (MSCs) are an essential precursor to bone marrow adipocytes and osteoblasts. The balance between this progenitor pool and mature cells (adipocytes and osteoblasts) is often skewed by disease and aging. In multiple myeloma (MM), a cancer of the plasma cell that predominantly grows within the bone marrow, as well as other cancers, MSCs, preadipocytes, and adipocytes have been shown to directly support tumor cell survival and proliferation. Increasing evidence supports the idea that MM-associated MSCs are distinct from healthy MSCs, and their gene expression profiles may be predictive of myeloma patient outcomes. Here we directly investigate how MM cells affect the differentiation capacity and gene expression profiles of preadipocytes and bone marrow MSCs. Our studies reveal that MM.1S cells cause a marked decrease in lipid accumulation in differentiating 3T3-L1 cells. Also, MM.1S cells or MM.1S-conditioned media altered gene expression profiles of both 3T3-L1 and mouse bone marrow MSCs. 3T3-L1 cells exposed to MM.1S cells before adipogenic differentiation displayed gene expression changes leading to significantly altered pathways involved in steroid biosynthesis, the cell cycle, and metabolism (oxidative phosphorylation and glycolysis) after adipogenesis. MM.1S cells induced a marked increase in 3T3-L1 expression of MM-supportive genes including *Il-6* and *Cxcl12* (SDF1), which was confirmed in mouse MSCs by qRT-PCR, suggesting a forward-feedback mechanism. *In vitro* experiments revealed that indirect MM exposure prior to differentiation drives a senescent-like phenotype in differentiating MSCs, and this trend was confirmed in MM-associated MSCs compared to MSCs from normal donors. In direct co-culture, human mesenchymal stem cells (hMSCs) exposed to MM.1S, RPMI-8226, and OPM-2 prior to and during differentiation, exhibited different levels of lipid accumulation as well as secreted cytokines. Combined, our results suggest that MM cells can inhibit adipogenic differentiation while stimulating expression of the senescence associated secretory phenotype (SASP) and other pro-myeloma molecules. This study provides insight into a novel way in which MM cells manipulate their microenvironment by altering the expression of supportive cytokines and skewing the cellular diversity of the marrow.

## Introduction

The pathogenesis of multiple myeloma (MM) involves bidirectional interactions of MM cells with bone marrow (BM) resident cells. MM cells often depend on these host cells to provide factors that aid in drug resistance and proliferation. Specifically, MM cells interact with osteoblasts (1–3), osteoclasts (4, 5), osteocytes (6), BM mesenchymal stem cells (MSCs) (7), and bone marrow adipocytes (BMAds) (8, 9), each playing a unique type of supportive role for MM cells. MSCs are a common progenitor for pericytes, osteoblasts, osteocytes, and adipocytes. The differentiation capacity of MSCs is influenced and regulated by many growth factors, canonical WNT signaling (10), and metabolic programming (11). Indeed, high fat diet in mice (11) and obesity in humans (12) have recently been shown to modulate the number of adipocyte progenitors in the marrow, skewing the delicate balance between MSCs, osteoblasts, and BMAds. Interestingly, obesity is also a risk factor for MM development and progression, with obese patients being 20% more likely than non-obese patients to transition from a premalignant stage, monoclonal gammopathy of undetermined significance (MGUS), to overt MM (13, 14). Obesity likely contributes to MM in many ways; for example, Bullwinkle et al. found that conditioned media from white adipocytes from obese patients contained increased IL-6, and when cultured with MM cells, led to increased MM cell survival and adhesion *via* increased STAT-3 (15). Lwin et al. found that diet-induced obesity increased IGF1 levels in mice and created a permissive BM microenvironment for the progression of MM from MGUS (16). Increased levels of BMAds have also been correlated with obesity in human patients (17), suggesting that obesity-associated levels of increased BMAds, likely contribute to an optimal MM microenvironment. Moreover, MM incidence increases with age, and BMAds make up 0% of BM cells in infancy and almost 70% in the elderly (18), again demonstrating a correlation between increased BMAds and MM. Thus, understanding the role of BMAds in MM, and how MM influences BMAds and their progenitors, is crucial for fully understanding MM disease progression and incidence.

The bidirectional relationship between myeloma cells and BMAds is yet to be fully elucidated; however, the data suggest that BMAds typically support myeloma cells. BMAds were previously believed to be inert bystanders, but in recent years, they were found to be intricate and responsive players in the BM microenvironment. BMAds contribute to systemic metabolism (19), bone remodeling (20), and hematopoiesis (21). Several studies have shown that BMAds influence MM cell proliferation, apoptosis, migration, and homing to the marrow (18, 22). Adipocyte-derived factors such as MCP-1/*CCL2* and SDF1α/*CXCL12* are chemotactic factors for myeloma cells (8, 15), while other adipokines promote myeloma proliferation (e.g., leptin/LEP) (18) and resistance to chemotherapies (e.g., leptin/LEP, adipsin/CFD) (9, 23). Recent studies have demonstrated that BMAds are modulated by MM cells (22, 24–26) and that MM-reprogrammed BMAds contribute to myeloma-induced bone disease (27). Myeloma patient-derived MSCs (MM-MSCs) also have alterations in the expression of transcripts involved in MM disease pathogenesis (IL-6) (28) as well as impaired osteogenic capabilities (7, 28–30). Evidence suggests that MM-MSCs have senescent characteristics accompanied by an aberrant secretory profile that may impair bone formation (7, 28, 30). Here we further investigate the adipogenic capacity of patient-derived MM-MSCs and model MM-induced changes in adipogenic progenitors with a co-culture system.

## Material and Methods

### Cell Culture

3T3-L1, human BM MSCs from normal, non-malignant bone marrow (NBM-MSCs), or myeloma patient bone marrow (MM-MSCs) (7), and naïve mouse BM MSCs (mMSCs) were cultured and differentiated as previously described (6). mMSCs were extracted from wild-type mice of C57BL6/J background of approximately 2–3 months of age. All experimental studies and procedures involving mice were performed in accordance with approved protocols from the Maine Medical Center Research Institute (Scarborough, ME, USA) Institutional Animal Care and Use Committee (IACUC), Reagan Laboratory protocol #1812. NBM-MSCs were isolated and utilized for experiments as previously described (31). 5TGM1, MM.1S, RPMI-8226, and OPM-2 cells were cultured as previously described (22, 31). For transwell co-culture experiments, stromal cells were seeded into the bottom of 6- or 24-well plates prior to adipogenic differentiation, allowed to adhere, and grown to 80–100% confluence depending on the cell type. Myeloma cells were then seeded either directly, or into the top of 0.4 µm transwell membranes (Corning; Corning, NY) and cultured for 48 h for indirect co-culture experiments, or allowed to remain in direct co-culture throughout adipogenic differentiation. Lipid droplets from adipocytes *in vitro* were labeled with Oil Red O alone, or in combination with DAPI (Thermo Fisher Scientific, Waltham, MA) and AF488-phalloidin (Invitrogen, Carlsbad, CA) and analyzed as previously described (6). All cell culture reagents were acquired from VWR unless stated.

### Total mRNA Extraction and Quantitative Reverse Transcriptase Polymerase Chain Reaction

Total RNA was harvested in QIAzol and prepared *via* Qiagen miRNEASY Kit with DNase On-column digestion (Qiagen; Hilden, Germany) according to the manufacturer’s protocol. Ribolock (1U/µl; Thermo Fisher Scientific) was added to inhibit RNA degradation in samples processed for microarray. mRNA was quantified and tested for quality and contamination using a Nanodrop 2000 (Thermo Fisher Scientific) and subjected to quality control minimum standards of 260/230 > 2 and 260/280 > 1.8 prior to downstream applications. qRT-PCR experiments were carried out as previously described (6). Ranges of Cq values utilized for qPCR experiments include human mesenchymal stem cells (hMSCs): *ACTB* (22.08–30.84), *PPARG* (25.80–34.45), *CEBPA* (27.62–35.03), *FABP4* (22.30–39.51); mBMSCs: *Actb* (17.56–19.25), *Pparg* (23.48–25.71), *Cebpa* (24.44–27.8), *Fabp4* (18.30–28.81), *Cxcl1* (27.50–34.40), *Cxcl2* (25.03–34.79), *Il6* (32.46–37.57); 3T3L1: *Actb* (18.37–38.15), *Pparg* (22.39–39.05), *Adipoq* (21.09–38.30), *Cxcl12* (19.45–37.30), *Cxcl1* (27.79–39.36), *Il6* (30.09–39.04).

### 3T3-L1 Gene Expression Assessed by Microarray

Total RNA (100 ng) was used for cRNA synthesis, prepared, and purified as previously described (22); 5.5 µg of fragmented single-strand cDNA (GeneChip ® WT PLUS reagent Kit) was purified, labeled, and hybridized prior to injection into Mouse Clariom S arrays, also as previously described (22). Arrays were placed in the Affymetrix® GeneChip® Hybridization Oven 645 and stained with the Affymetrix GeneChip® Fluidics Station 450 prior to scanning (7G Affymetrix GeneChip Scanner 3000). Raw data (Affymetrix CEL files) were imported into the Gene Expression Workflow (Partek Genomics Suite v. 6.17.0918, Partek, St Louis, MO) (GSE143269) and normalized prior to log2 transformation, and differential expression (DE) analysis, as previously described (22), except here a one-way ANOVA was utilized in the DE analysis and DE genes were defined based on an absolute fold change > 1.5 in combination with an unadjusted p-value of 0.05 or less.

Differential expression of functional groups was assessed through Pathway-ANOVA and GO-ANOVA analyses in Partek Genomic Suite, which utilized Kyoto Encyclopedia of Genes and Genomes (KEGG) Pathway and GO term databases, respectively. Both Pathway- and GO-ANOVA were performed on normalized expression data without filtering using the method of moments algorithm. Parameters for the Pathway- and GO-ANOVA analyses were configured such that only pathways with more than two and fewer than 500 genes are considered and only GO-terms with more than two and fewer than 100 genes were considered.

Normalized gene expression values were also subjected to a gene set enrichment analysis (GSEA) using the Java implementation from the Broad Institute (32). Array probe sets were collapsed into gene symbols for the analysis and the chip platform used for annotation was Clariom_S_Mouse.r1.chip available from the annotation directory within the GSEA software. Phenotype labels for treatment (the factor of interest) were “MM.1S” and “Control.” “Diff_of_Classes” was used as the gene ranking metric. Several Molecular Signatures Database Collections (MSigDB v6.1, Dec 2, 2017) were used to identify gene sets significantly enriched in MM.1S vs control cultures for both the preadipocyte and mature adipocyte experiments, including H (Hallmark), C2 (curated gene sets), and C5 (Gene Ontology, GO, gene sets), which contained 50, 3,689, and 4,429 gene sets respectively, when gene set size filters min=15 and max=500 were applied (33). Only those gene sets with a false discovery rate (FDR) < 25% were considered significantly enriched.

### Analysis of External Multiple Myeloma-Mesenchymal Stromal Cells and Normal Bone Marrow-Mesenchymal Stromal Cells Dataset

Microarray gene expression data from Corre et al. (28) was accessed for MSCs derived from non-malignant, normal bone marrow (NBM; n=7) or multiple myeloma (MM; n=6) patient bone marrow. Differentially expressed (DE) genes were determined *via* unpaired, two-tailed t-test for each transcript (p<0.05). Gene relatedness was assessed with a tool for recurring instances of neighboring genes (STRING v11.0), input was limited to significant DE genes with a fold-change ≥|2| (34). Heatmaps were generated using the publicly available Morpheus data visualization tool through the Broad Institute (https://software.broadinstitute.org/morpheus/).

### Analysis of p16 and p21 in Adipogenic Precursors and Adipocytes

Protein from cell lysates was extracted using RIPA buffer (Santa Cruz, 24948) and quantified using DC protein assay kit II (Bio-Rad, 5000112). Each sample was denatured in 4x laemmli buffer (Bio-Rad, 1610747) for 5 minutes at 95°C. Samples were run on 12% polyacrylamide gels (Bio-Rad, 5671043) and transferred onto PVDF membranes (Bio-Rad, 1704156). Primary antibodies anti-p16 (Abcam, ab108349; 5% milk in TBS-T, 3 days), p21 Waf1/Cip1 (Cell Signaling Technology, #2947; 5% BSA in TBS-T, 1 day) and β-tubulin (Cell Signaling Technology, #2146; 5% BSA in TBS-T, 1 day) were used at a 1:1,000 dilution with incubation at 4°C. HRP-linked anti-rabbit IgG (Cell signaling technology, #7074; 5% BSA in TBS-T) was used as the secondary antibody at a 1:5,000 dilution with incubation at 4°C for 24 h.

### Analysis of Secreted Cytokines Following Exposure to Multiple Myeloma Cells

Cell culture conditioned media (CM) was collected from mature naïve adipocytes or MM-adipocytes in culture after 48 (mouse) or 72 (human) hours of incubation and frozen at −20°C. Secreted cytokines in the CM were quantified with either the Mouse Cytokine Array A (R&D; Minneapolis, MN) or the Human Cytokine Array (R&D) per the manufacturer’s instructions.

### *In Vitro* Myeloma Cell Functional Characterization

Myeloma cell number was assessed using bioluminescence (MM.1S, OPM-2) or CellTiter-Glo (5TGM1, RPMI-8226; Promega, City, State). Cells were collected and stained with APC-Annexin V (BioLegend, San Diego, CA) and DAPI (Thermo Fisher Scientific, Waltham, MA) for apoptosis. For proliferation, cells were fixed in fixation buffer (BioLegend) prior to washing and staining with Alexa Fluor 647 anti-human Ki-67 antibody (BioLegend) prior to flow cytometry *via* MACSQuant Analyzer (Miltenyi Biotec, Bergisch Gladbach, Germany). A minimum of 10,000 events were captured and analyzed using FlowJo v.10.1 (Becton, Dickinson & Company, Ashland, OR).

### Statistical Analysis

All graphs were created with GraphPad Prism (version 7); statistical significance was determined by using two-way ANOVA (multiple groups) or Student’s T-test (two groups) unless otherwise stated. For these tests, we made the assumptions that the data had a Gaussian distribution and that they meet the qualifications for a parametric test (normality, equal variance, and independence). All data are expressed as mean ± SEM. For more information, please reference *Supplemental Methods*. Statistical analyses for the microarray data were completed as outlined above.

## Results

### Multiple Myeloma Patient-Derived Mesenchymal Stromal Cells Exhibit Changes in Key Metabolic Genes

We began with a thorough analysis of the publicly available data from Corre et al. (28), investigating relative differences in the gene expression profiles in MSCs from normal bone marrow (NBM) and multiple myeloma (MM) patient bone marrow. We found 224 downregulated genes in MM-MSCs compared to NBM-MSC controls, and 183 of these genes were connected nodes in our gene network analysis (STRINGv11.0, **Supplementary Figure 1A**). In the significantly downregulated genes, we observed significant enrichment of transcripts encoding genes in the cellular differentiation pathway (GO:0030154; FDR<0.05) including the transcription factors forkhead box transcription factors A1 and M1 (*FOXA1*, *FOXM1*), pioneering transcription factors that can enhance or suppress the expression of differentiation and/or proliferation factors (35, 36) as well as lim homeobox 8 (*LHX8*), which is involved in the pro-osteogenic BMP signaling cascade (37). Nine genes specifically involved in osteogenesis were also significantly downregulated including collagen type XI alpha 1 chain (*COL11A1*), the BMP4 antagonist chordin like 1 (*CHRDL1*), insulin like growth factor 1 (*IGF1*), and asporin (*ASPN*) which binds collagen and calcium in cartilage, inducing cartilage mineralization. The leptin receptor (*LEPR*), a known regulator of osteogenic and adipogenic progenitors (38), was also significantly decreased in MM-MSCs compared to controls, suggesting that in addition to impaired osteogenic capacity, as previously described (28), adipogenic capacity may also be impaired in MM-MSCs.

**Figure 1:**
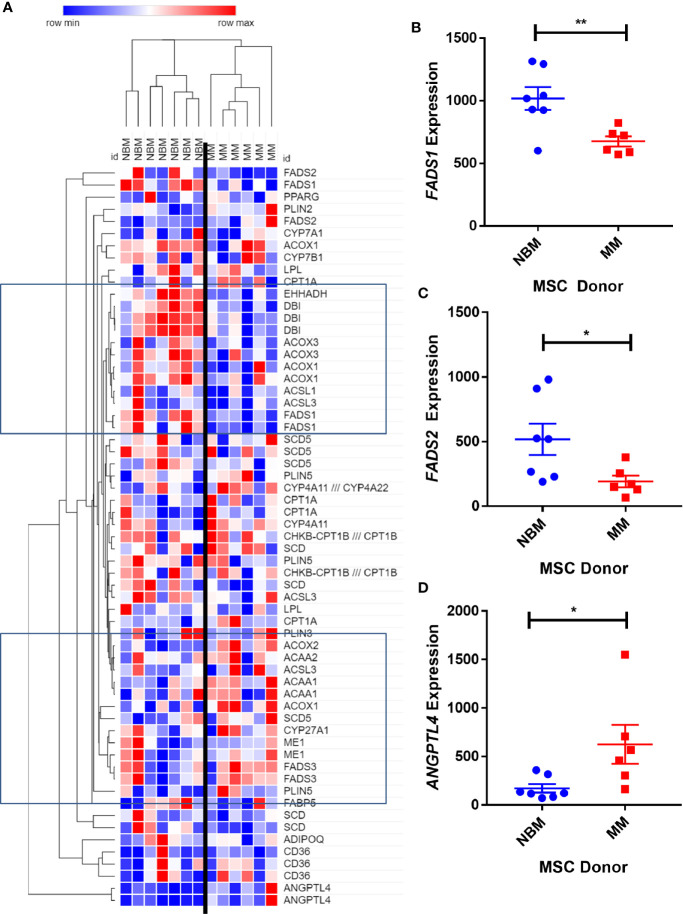
Expression of genes encoding key metabolic proteins in myeloma patient mesenchymal stromal cells (MSCs). Expression of transcripts involved in PPAR signaling Kyoto Encyclopedia of Genes and Genomes (KEGG) pathway **(A)**, in MSCs derived from normal patient bone marrow (NBM) or myeloma patients (MM), graphically demonstrated using Morpheus software (The Broad Institute). Groups of down and upregulated genes can roughly be seen in the top and bottom boxed regions. Reduced gene expression of *FADS1* **(B)** and *FADS2* **(C)** and increased expression of *ANGPTL4* **(D)**. Analysis of publicly available data from Corre *et al*. 2007, *Leukemia*.

**Figure 2:**
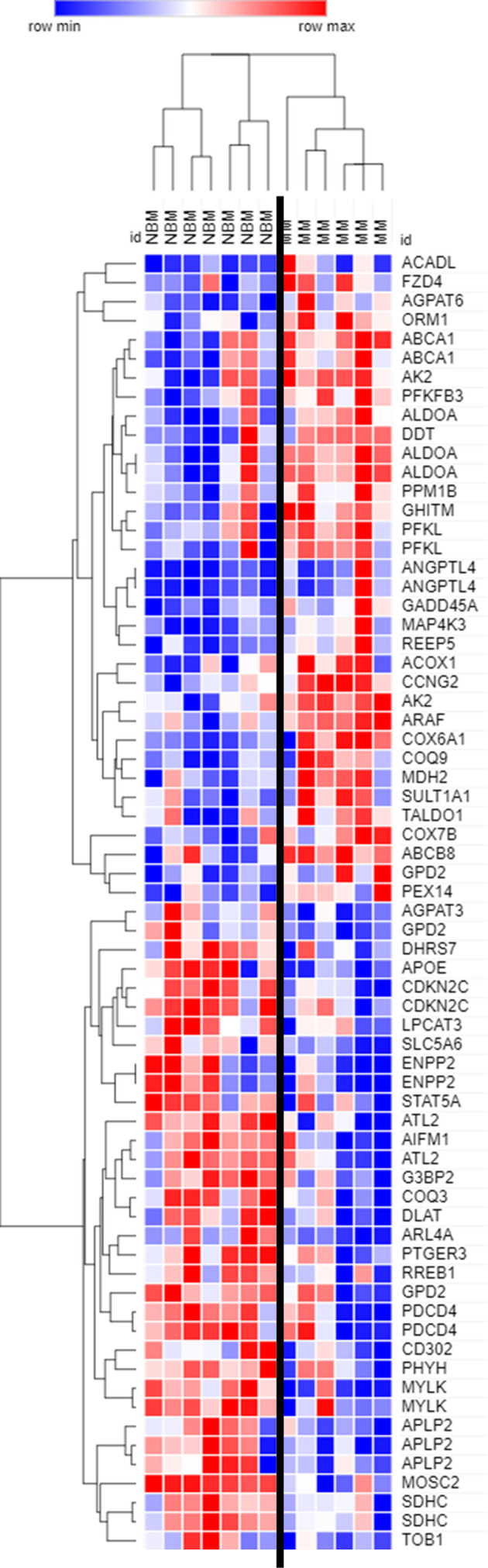
Expression of genes encoding key adipogenic proteins in myeloma patient mesenchymal stromal cells (MSCs). Expression of transcripts included in the Hallmark Adipogenesis Gene Set, in MSCs derived from normal patient bone marrow (NBM) or myeloma patients (MM). Graphically demonstrated using Morpheus software (The Broad Institute). Analysis of publicly available data from Corre *et al*. 2007, *Leukemia*.

We next created two clusters of transcripts involved in both the KEGG PPAR signaling pathway (**Figure 1A**) and the Hallmark adipogenesis gene set (**Figure 2**) to begin to specifically investigate the effects of MM exposure on lipid metabolism and metabolic homeostasis. Within the PPAR signaling pathway, we observed significantly altered expression of 14 transcripts (associated with nine genes) within this cluster (**Table 1**). Seven out of nine genes were downregulated, including fatty acid desaturase 1 (*FADS1*; **Figure 1B**, **Supplementary Figure 1A**) and fatty acid desaturase 2 (*FADS2*; **Figure 1C**), potentially representing a modulation of the processing of long-chain polyunsaturated fatty acids in MM-MSCs. MM-MSCs exhibited slight but significant increases in acetyl-coA acyltransferase 1 (*ACAA1*), acyl-CoA oxidase 1 (*ACOX1*), and acyl-CoA oxidase 2 (*ACOX2*), which are involved in fatty acid oxidation. These findings led us to hypothesize that MM cells modulate the metabolism of MSCs and may alter their adipogenic differentiation. As previously reported by Corre *et al*., we also confirmed >2 fold increases in two transcripts for angiopoietin like 4 (*ANGPTL4*; **Figure 1D**, **Supplementary Figure 1B**), the expression of which is responsive to peroxisome proliferation activators (28).

**Table 1:** 14 Metabolic genes significantly altered in myeloma patient-derived mesenchymal stem cells (MM-MSCs).

Gene symbol	Gene name	P-value	Relative expression (MM/NBM)
*EHHADH*	Enoyl-CoA hydratase and 3-hydroxyacyl CoA dehydrogenase	0.0024	0.64
*DBI*	Diazepam binding inhibitor, acyl-CoA binding protein	0.0028	0.80
*FADS1*	Fatty acid desaturase 1	0.0030	0.57
*DBI*	Diazepam binding inhibitor, acyl-CoA binding protein	0.0037	0.81
*DBI*	Diazepam binding inhibitor, acyl-CoA binding protein	0.0045	0.78
*ACOX2*	Acyl-CoA oxidase 2	0.0046	1.17
*FADS1*	Fatty acid desaturase 1	0.0083	0.66
*FADS1*	Fatty acid desaturase 1	0.0143	0.48
*ACOX3*	Acyl-CoA oxidase 3, pristanoyl	0.0300	0.75
*ANGPTL4*	Angiopoietin like 4	0.0355	2.68
*ANGPTL4*	Angiopoietin like 4	0.0365	3.63
*FADS2*	Fatty acid desaturase 2	0.0384	0.37
*ACAA1*	Acetyl-CoA acyltransferase 1	0.0447	1.17
*ACOX1*	Acyl-CoA oxidase 1	0.0452	1.35

Analysis of publicly available data from Corre et al. 2007, Leukemia; p<0.05. Blue text indicates downregulated; red indicates upregulated.

By further examining genes that were significantly different (p<0.05) between MM and NBM-MSCs in the Corre dataset, we observed differential expression of 68 genes that are specifically upregulated during adipogenesis (**Figure 2**). The cluster of genes upregulated in MM-MSCs included acyl-CoA dehydrogenase long chain (*ACADL*)—a mitochondrial enzyme involved in fatty acid metabolism, as well as aldolase fructose-bisphosphate A (*ALDOA*), a glycolytic enzyme, suggesting that MM-MSCs burn rather than store more fatty acids than normal MSCs. ATP binding cassette subfamily A member 1 (*ABCA1*), a membrane-associated protein involved in cellular lipid removal, was also upregulated in MM-MSCs. The cluster of transcripts downregulated in MM-MSCs included metabolic enzymes 1-acylglycerol-3-phosphate O-acyltransferase 3 (*AGPAT3*), ectonucleotide pyrophosphatase/phosphodiesterase 2 (*ENPP2*), and dihydrolipoamide S-acetyltransferase (*DLAT*). Signal transducer and activator of transcription 5A (*STAT5A*), a key molecule involved in the signaling cascades triggered by many ligands including interleukins and growth hormones, was also downregulated in MM-MSCs compared to controls. Interestingly, key regulators of cell cycle and apoptosis were downregulated in MM-MSCs, including cyclin dependent kinase inhibitor 2C (*CDKN2C*), a cell cycle regulator that controls G1 progression, and programmed cell death 4 (*PDCD4*), which regulates proliferation by inducing cell cycle arrest at G1 (39). Overall, these data suggest that MM patient derived MSCs exhibit changes in key metabolic genes that may inhibit their ability to differentiate into adipocytes.

In a second study utilizing mesenchymal cells isolated from patient bone marrow biopsies (40), MM-MSCs exhibited differential expression profiles compared to ND-MSCs. The authors identified seventy-eight differentially expressed genes between MM- and ND-MSCs, with transcripts representing processes such as cell adhesion, cell cycle and proliferation, and transcriptional regulation. We utilized the data from this study to investigate functional enrichments and connectedness of the DE genes (**Supplementary Figure 2**). Our analysis revealed that 31 of the 78 genes (approximately 40% of the differentially expressed genes) were involved in the regulation of cellular metabolic process (GO:0031325), including a number of transcription factors (**Supplementary Figure 2A**) including nuclear receptor subfamily 1, group D, member2 (NR1D2), SRY-box transcription factor 9 (SOX9), nuclear receptor subfamily 2 group F member 1 (NR2F1), paired like homeodomain 2 (PITX2), and YY1 transcription factor (*YY1*). *ENPP1* was also significantly decreased in MM-MSCs compared to controls (**Supplementary Figure 2B**), as we have highlighted above in the dataset from Corre et al. In addition, in this second study MM-MSCs exhibited significantly decreased expression of CCAAT enhancer binding protein alpha (*CEBPA*; **Supplementary Figure 2B**), which functions as a key early adipogenic transcription factor. In addition, *SOX9* and *MEIS1* were upregulated in MM-MSCs vs. NBM-MSCs (**Supplementary Figure 2C**), but the inactivation of these two factors have been linked to adipogenic differentiation (41). In addition, zinc finger protein 36 (*ZFP36*) and Salvador family WW domain containing protein 1 (*SAV1*), which have been linked to positive regulation of fat cell differentiation (GO:0045600) were also downregulated in MM-MSCs.

### Primary Mesenchymal Stromal Cells From Mouse and Human Bone Marrow Exhibit Reduced Adipogenic Gene Expression Profiles During the Differentiation Process

**Figure 3:**
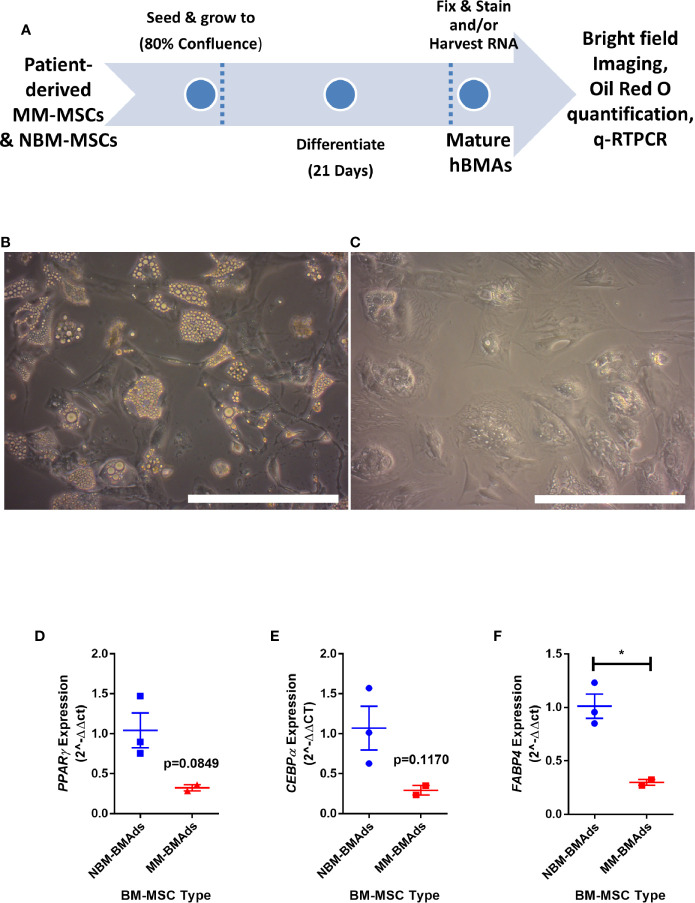
Mesenchymal stromal cells (MSCs) derived from myeloma patients exhibit impaired adipogenic differentiation. Experimental design of MSC differentiation experiment **(A)**. MSCs differentiated into adipocytes for 21 days from normal donor bone marrow (NBM-BMAds), **(B)**, or myeloma patient bone marrow (MM-BMAds, **(C)** for 21 days; images taken at 20X, scale bars = 250 µm. *PPARG* **(D)**, *CEBPA* **(E)**, and *FABP4* **(F)** expression are decreased at the end of the adipogenic differentiation period in adipocytes derived from myeloma patient MSCs (MM-BMAds), relative to normal donor controls (NBM-BMAds) (n=3); *p < 0.05.

To determine if the gene expression changes above translated to functional effects, we next tested our hypothesis that adipogenesis is inhibited in MM-MSCs by comparing the *in vitro* differentiation capacity of normal bone marrow (NBM) and myeloma donor (MM) MSCs (**Figure 3A**). While NBM-MSCs readily differentiated into adipocytes (**Figure 3B**), MM-MSCs exhibited diminished differentiation capacity (**Figure 3C**; **Supplementary Figure 2A**). Having observed phenotypic differences in differentiated MM-MSCs, we next tested their relative gene expression of key adipogenic transcripts by qRT-PCR and observed suppression of *PPARG* (**Figure 3D**), *CEBPA* (**Figure 3E**), and *FABP4* (**Figure 3F**). These findings demonstrate significantly reduced adipogenic capacity of MM-MSCs compared to NBM-MSCs using patient stromal cells.

**Figure 4:**
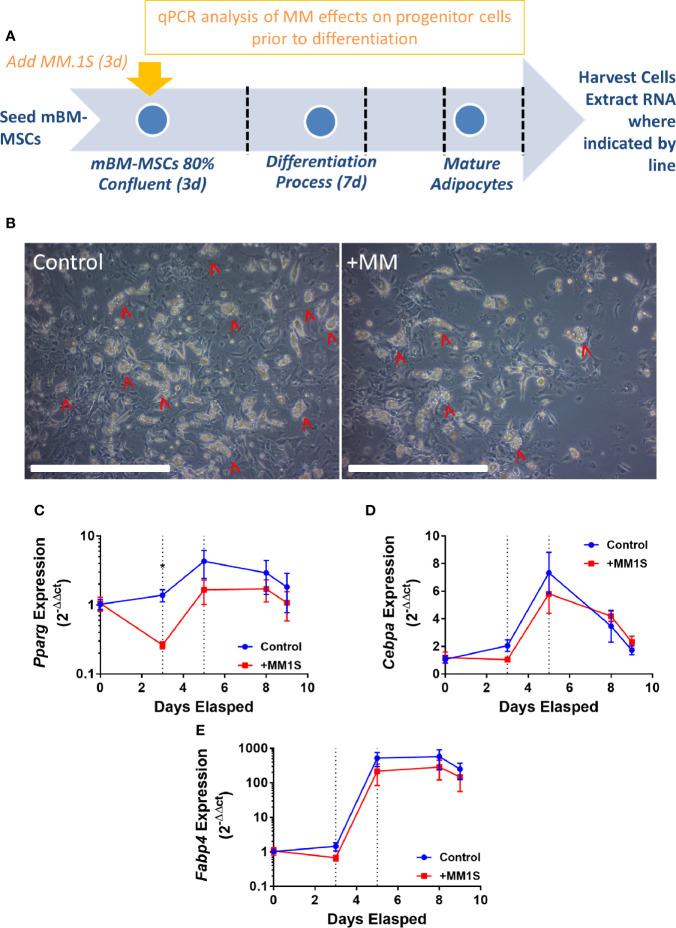
Mouse bone marrow mesenchymal stem cells (BM-MSCs) exposed to multiple myeloma (MM) cells *via* indirect co-culture *in vitro* exhibit reduced adipogenesis. Experimental design of co-culture experiment **(A)**. Images taken with 10X objective (scale bars = 500 µm) at terminal differentiation following pre-exposure (day 9; **B**). *Pparg* **(C)**, *Cebpa* **(D)**, and *Fabp4* **(E)** expression as assessed by qRT-PCR is suppressed in mouse MSCs “pre-exposed” to myeloma cells *in vitro* for 72-h *prior to differentiation* (first dotted line indicates the end of pre-exposure and start of differentiation), and levels are slightly suppressed throughout differentiation (second dotted line represents day 2 of adipogenic differentiation) (n=3); *p ≤ 0.05.

This led to us to ask if MM cells themselves directly cause these alterations in MSCs, thus, we next tested the hypothesis that MM cells can directly induce changes in primary BM stromal cells with *in vitro* co-culture. Primary BM stromal cells from naïve mice (**Supplementary Figure 2B**) were exposed to MM.1S myeloma cells *via* transwell co-culture for 72 h prior to adipogenic differentiation (**Figure 4A**). Imaging after differentiation revealed significantly fewer lipid-containing adipocytes in cultures with MM pre-exposure, compared to controls (**Figure 4B**). Immediately after MM exposure (day 3), and 2 days later (day 5), mouse MSCs exhibited suppression of the key adipogenic transcription factor *Pparg* (**Figure 4C**) with slight, but non-significant suppression of both *Cebpa* (**Figure 4D**) and the mature adipocyte marker *Fabp4* (**Figure 4E**). While expression levels of these adipogenic transcripts were reduced, the overall pattern of induction mirrored that of the control cells, suggesting that adipogenic differentiation was occurring at some capacity. These experiments support the hypothesis that MM cells induce rapid and long-lasting effects on adipocyte precursors, and that these effects are at least in part mediated through soluble molecules.

### Indirect Co-Culture with Myeloma Cells Results in Reduced Lipid Accumulation and Inhibition of Adipogenic Transcripts in Differentiating Mouse Preadipocytes

**Figure 5:**
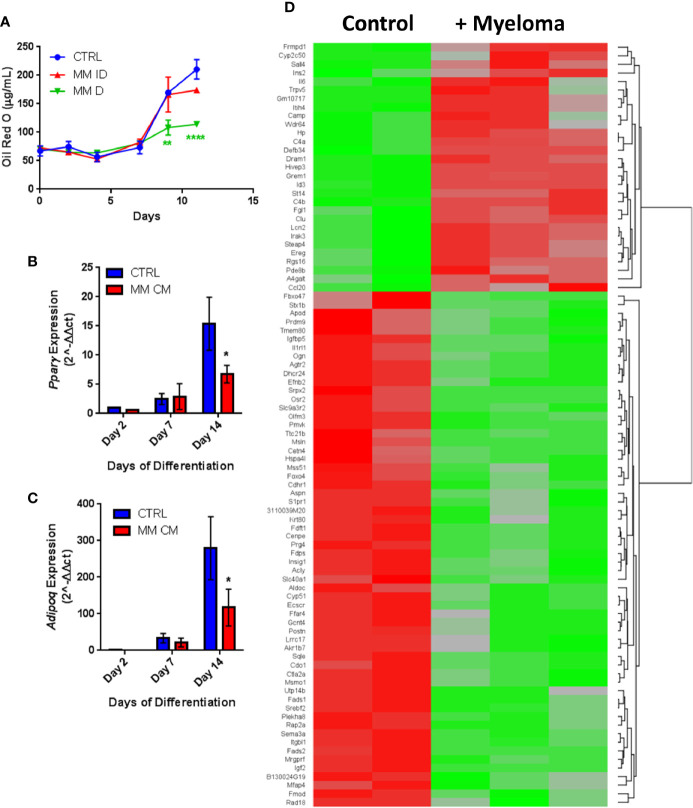
Adipogenic differentiation of 3T3-L1 preadipocytes is inhibited by MM.1S myeloma cells. 3T3-L1 adipocytes were assessed for Oil Red-O content either alone (control) or with exposure to MM.1S cells prior to differentiation process *via* indirect (+MM ID) or direct (+ MM D) co-culture (day 11). Lipid content is significantly reduced by MM.1S co-culture (indirect, MM ID; direct, MM D) *during* differentiation compared to 3T3-L1 cells on their own (CTRL); quantification of Oil Red-O staining **(A)**. Expression of adipogenic transcripts *Pparg* **(B)** and *Adipoq* **(C)** are suppressed *during* differentiation in the presence of conditioned media from MM.1S cells; n=4. Significant differences in gene expression (p ≤ 0.05, FC |1.8|, red is upregulated, green is downregulated) in 3T3-L1 adipocytes exposed to myeloma cells for 48-h *prior* to differentiation as measured by microarray **(D)**; control n=2, MM.1S n=3; *p < 0.05, **p < 0.01, ****p < 0.0001.

Having observed similar effects in primary human samples and *in vitro* with co-cultures, we next investigated MM-induced changes in adipogenic precursors using the 3T3-L1 murine preadipocyte cell line, to eliminate donor variability. We utilized multiple different experimental designs to characterize the effects of MM cells on 3T3-L1 cells (MM-3T3-L1s): pre-exposure of 3T3-L1 cells to MM cells for 2 days *prior to* differentiation (using direct or indirect co-cultures; **Supplementary Figure 4A**), and co-culture of 3T3-L1 cells with MM cells *during* differentiation (through exposure to MM conditioned media (MM-CM) during differentiation; **Supplementary Figure 4B**). 3T3-L1 preadipocytes exposed to MM.1S cells during differentiation exhibited significantly reduced lipid content in direct co-culture and this trend also seen in indirect co-culture (**Figure 5A**). Similarly, 3T3-L1 cells exposed to MM-CM during differentiation exhibited significantly reduced *Pparg* gene expression (**Figure 5B**), and adiponectin, *Adipoq* (**Figure 5C**) at day 14. Although we observed these reductions with MM co-culture, lipid accumulation did increase over the course of differentiation, and the expression of adipogenic transcripts was also turned on, suggesting that MM cells inhibit, but do not completely block adipogenesis.

Indirect exposure of 3T3-L1 preadipocytes to MM.1S cells *via* transwell for 48 h prior to differentiation (**Supplementary Figure 4A**), revealed significant differences in the expression of 1287 total genes as assessed by microarray (**Figure 5D**). Of these, 105 transcripts were significantly upregulated (>1.5-fold, p<0.05; **Supplementary Table 1**) and 179 were significantly downregulated (<1.5-fold, p<0.05; **Supplementary Table 2**). Among them, we observed increased expression of interleukin-1-receptor- associated kinase 3 (*Irak3*) and lipocalin 2 (*Lcn2*) (**Table 2**; **Supplementary Table 1**), a small transport protein involved in the shuttling of small hydrophobic molecules. We also detected decreased expression of insulin growth factor (*Igf2;* **Table 2**; **Supplementary Table 2**), a growth factor which promotes subcutaneous preadipocyte differentiation (42). Also, of note, we detected significantly reduced expression of odd-skipped related 2 (*Osr2*), which has been linked to the control of BMP and semaphorin 3A (SEMA3A) signaling. Interestingly, *Sema3a* was also suppressed in MM-3T3-L1s (**Table 2**; **Supplementary Table 2**), and its expression is known to inhibit MM progression in mouse models (43). Overall, we have seen that MM cells reduce lipid accumulation and inhibit adipogenic transcripts in differentiating mouse preadipocytes.

**Table 2:** Altered expression of transcripts in 3T3-L1 adipocytes with exposure to MM.1S cells.

Increased in adipocytes w/pre-exposure to MM cells	p-value	Fold change (MM/CTRL)	Decreased in adipocytes w/pre-exposure to MM cells	P-value	Fold change (MM/CTRL)
Cathelicidin antimicrobial peptide *(Camp)*	0.046908	*3.725*	Angiotensin II receptor, type 2 *(Agtr2)*	0.002039	−9.069
**Interleukin-1 receptor associated kinase 3 *(Irak3)***	0.011152	*3.644*	Aldolase C, fructose-bisphosphate *(Aldoc)*	0.012447	−4.186
CD4 antigen *(C4a)*	0.004946	*2.405*	Interleukin 1 receptor-like 1 *(Il1rl1)*	0.009467	−4.055
**Lipocalin 2 *(Lcn2)***	0.012884	*2.346*	Apolipoprotein D *(Apod)*	0.02907	−3.747
Complement component 4 b *(C4b)*	0.000832	*2.300*	**Semaphorin 3a *(Sema3a)***	0.001421	−3.282
Fibrinogen-like protein 1 *(Fgl1)*	0.01909	*2.241*	UTP14B small subunit processome component *(Utp14b)*	0.025423	−3.246
Inhibitor of DNA binding 3 *(Id3)*	0.00221	*2.195*	**Odd-skipped related 2 *(Osr2)***	0.002808	−3.191
Alpha 1,4, galctosyltransferase *(A4galt)*	0.047703	*2.181*	Cytochrome P450, family 51 *(Cyp51)*	0.005149	−3.089
Haptoglobin *(Hp)*	0.004879	*2.170*	**Insulin-like growth factor 2 *(Igf2)***	0.000666	−2.955
STEAP family member 4 *(Steap4)*	0.014702	*2.125*	Integrin, beta-like 1 *(Itgbl1)*	0.000345	−2.891

Top 10 most significant gene expression changes in 3T3-L1 adipocytes with exposure to MM.1S cells prior to differentiation derived from microarray data. Bold indicates discussed in text. Blue text indicates downregulated and red text indicates upregulated.

### Myeloma Cells Drive Aberrant Expression of Genes Involved in Critical Pathways in Mouse Adipocytes via Soluble Signals

**Figure 6:**
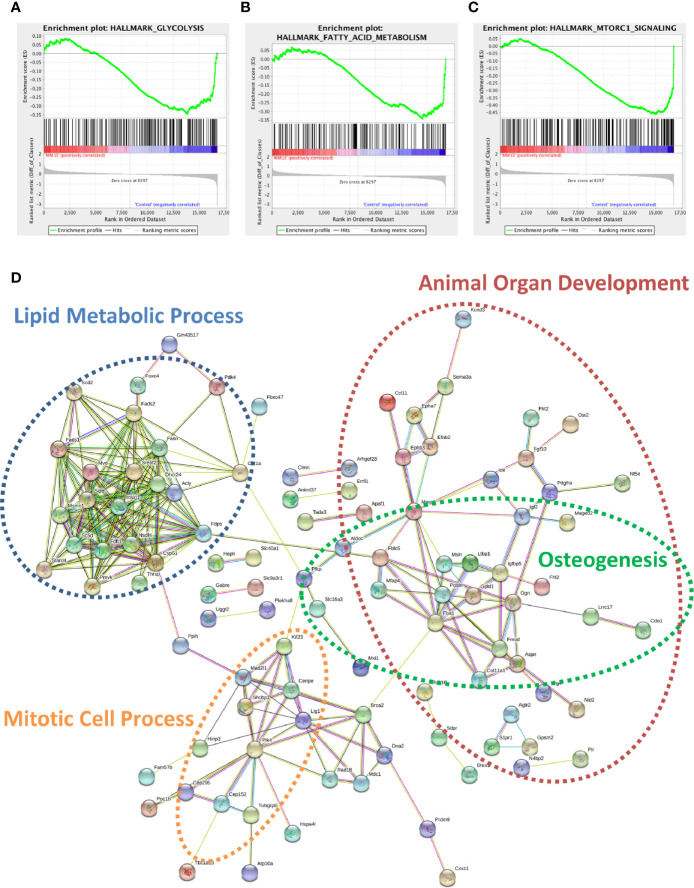
Cellular metabolism and key signaling pathways are altered in myeloma-associated mouse adipocytes. 3T3-L1 adipocytes exposed to MM.1S prior to differentiation have altered expression of genes involved with glycolysis **(A)**, fatty acid metabolism **(B)**, and mTORC signaling **(C)** as determined *via* gene set enrichment analysis (GSEA) analysis of 3T3-L1 microarray data. Expression of genes involved in lipid metabolism and encoding essential growth factors are downregulated in MM-3T3-L1 adipocytes **(D)** as visualized by string-db analysis of 3T3-L1 microarray data (p < 0.05, FC<−1.5).

Utilizing our indirect co-culture system with pre-exposure of 3T3-L1 cells to MM cells prior to differentiation (**Supplementary Figure 4A**), we next examined whether MM cells induce changes in major pathways when co-cultured with 3T3-L1 cells within the microarray data. The differential expression results from Pathway-ANOVA analysis of microarray data show several significantly enriched KEGG pathways including steroid biosynthesis and oxidative phosphorylation, cell cycle, DNA replication, and ubiquitin-mediated proteolysis (**Table 3**). Both the oxidative phosphorylation (**Table 3**) and glycolysis (not shown) KEGG pathways were enriched in 3T3-L1 adipocytes with pre-exposure to MM cells prior to differentiation, suggesting that exposure to MM-derived factors leads to metabolic dysfunction in preadipocytes and mature adipocytes. In addition to KEGG pathway analysis, we utilized normalized microarray data to perform gene set enrichment analysis (GSEA). While many signatures were not significant, GSEA analysis indicated significant enrichment (FDR<25%) in 14 out of 50 Hallmark gene sets (**Table 4**) including glycolysis (**Figure 6A**), fatty acid metabolism (**Figure 6B**), and mTORC signaling (**Figure 6C**).

**Table 3:** Microarray data reveal key cellular Kyoto Encyclopedia of Genes and Genomes (KEGG) pathways altered in response to multiple myeloma (MM) cell exposure.

KEGG pathway (pretreat)	P-value	FC
**Steroid biosynthesis**	5.84E−16	−1.41136
**Cell cycle**	1.67E−12	−1.08717
Terpenoid backbone biosynthesis	1.22E−08	−1.2398
**DNA replication**	1.23E−07	−1.13989
Rheumatoid arthritis	4.03E−07	1.08281
**Ubiquitin mediated proteolysis**	5.50E−07	−1.0509
Protein processing in endoplasmic reticulum	1.02E−06	−1.04289
**Oxidative phosphorylation**	1.46E−06	1.03672
Phagosome	2.37E−06	1.04667
Systemic lupus erythematosus	4.97E−06	1.05049

KEGG Pathways significantly altered in 3T3-L1 adipocytes with exposure to MM.1S cells prior to differentiation derived from microarray data. Pathway analyses incorporate all robust multi-array average (RMA)-normalized genes. Bold indicates discussed in text. Blue text indicates downregulated and red text indicates upregulated.

**Table 4:** Key gene sets revealed as altered in response to multiple myeloma (MM) cell exposure.

Hallmark gene set enriched	FDR (<0.25)
Androgen response	0.091
Mitotic spindle	0.091
**mTORC1 signaling**	0.091
Cholesterol signaling	0.091
G2M checkpoint	0.118
Protein secretion	0.122
Myogenesis	0.129
E2F targets	0.142
**Glycolysis**	0.148
Hypoxia	0.155
UV response DNA	0.155
**Fatty acid metabolism**	0.157
Apical junction	0.198
PI3K-AKT-MTOR signaling	0.206

Significant gene set enrichment analysis (GSEA) results in 3T3-L1 adipocytes with exposure to MM.1S cells prior to differentiation compared to controls. Derived from microarray data; analyses incorporate all robust multi-array average (RMA)-normalized genes. Bold indicates discussed in text.

Within the 179 significantly downregulated genes (**Supplementary Table 2**) in MM-3T3-L1s (**Figure 6D**), we observed enrichment in sterol, lipid, and cholesterol metabolic processes by gene network analysis- including 27 genes specifically linked to lipid metabolic processes. Like our data from analysis of normal donor versus myeloma patient MSCs (**Table 1**), we again found that *Fads1* and *Fads2* were significantly downregulated in MM-3T3-L1s compared to controls. Additionally, the expression of the key enzymes fatty acid synthase (*Fasn*) and carnitine palmitoyltransferase 1a (*Cpt1a*) were both suppressed in adipocytes with MM pre-exposure demonstrating altered metabolic activity, with specific implications for fatty acid synthesis and β-oxidation.

**Figure 7:**
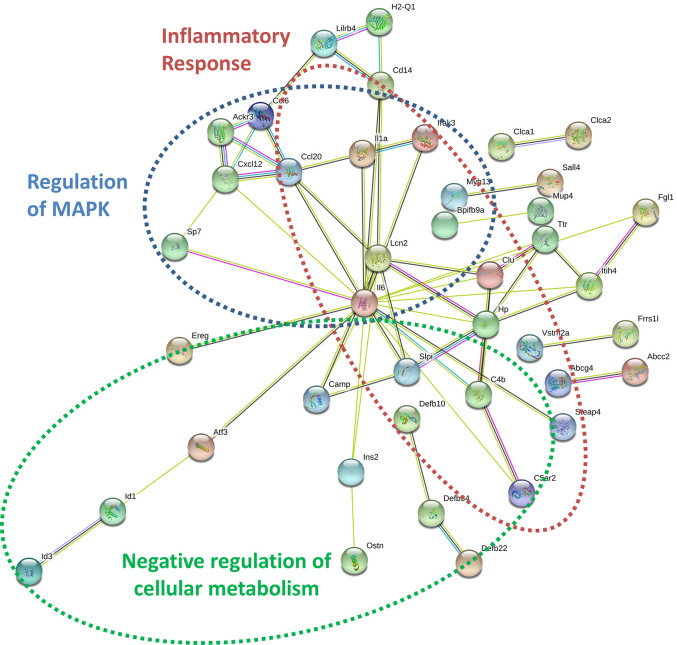
Key signaling pathways are altered in myeloma-associated mouse 3T3-L1 adipocytes. Upregulated genes in MM-3T3-L1s are connected *via* the central node of Il6 as demonstrated graphically by string-db analysis of 3T3-L1 microarray data.

By specifically examining the 103 significantly upregulated genes in 3T3-L1 adipocytes with prior exposure to MM cells, we further identified a network of genes with *Il6* as a central node (**Figure 7**). Collectively, these genes are enriched for GO terms such as “cellular response to interleukin-1” (GO:0071347), “inflammatory response” (GO:0006954), and “response to tumor necrosis factor” (GO:0034612). These results suggest that 3T3-L1 adipocytes exposed to MM cells in their progenitor stage increase the expression of genes that produce inflammatory proteins known to modulate the bone marrow microenvironment, and many of these are known members of the senescence-associated secretory phenotype (SASP).

### Multiple Myeloma Induces Expression of Key Transcripts Involved in the Senescence Associated Secretory Phenotype in Adipocyte Lineage Cells

**Figure 8:**
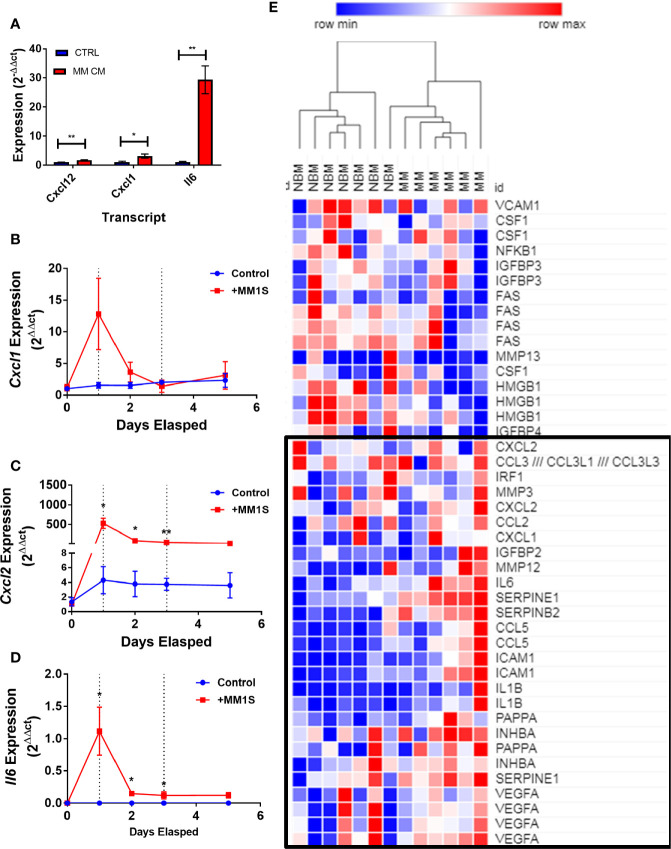
Exposure to myeloma cells prior to adipogenic differentiation induces SASP production in adipocyte precursors. 3T3-L1 cells exposed to MM.1S soluble factors during differentiation exhibit increased expression of: *Il6*, *Cxcl1*, and *Cxcl12* after terminal differentiation **(A)**. Mouse bone marrow MSCs were exposed to MM.1S cells *in vitro* for up to 72 h prior to adipogenic differentiation. Cells were harvested for RNA extraction after 24 (first dotted line), 48, and 72 (second dotted line; change to adipogenic media) hours of pre-exposure, and after the first treatment of adipogenic media (day 5). *Cxcl1* **(B)**, *Cxcl2* **(C)**, and *Il6* **(D)** gene expression was quantified in mMSCs at each time point; n=3 per group, *p < 0.05, **p < 0.01. Reanalysis of previously published MSC gene expression data comparing bone marrow from normal donors (NBM) and myeloma donors (MM) examining the SASP gene cluster **(E)** data from Corre *et al*. 2007, *Leukemia*.; heatmap generated *via* Morpheus.

Having obtained evidence that MM-3T3-L1s express genes encoding SASP proteins, we wanted to verify this in each of our cell types. We confirmed that *Cxcl12* and two traditional SASP genes, *Cxcl1*, and *Il6*, were elevated in MM-3T3-L1s *versus* control 3T3-L1s by qRT-PCR (**Figure 8A**). To confirm the presence of elevated SASP transcripts in primary cells, we first utilized mouse MSCs pre-exposed to MM.1S cells. After 24 h of MM exposure, we observed extremely high expression of *Cxcl1* (**Figure 8B**), *Cxcl2* (**Figure 8C**), and *Il6* (**Figure 8D**) which was not basally expressed in mouse BM stromal cells. Elevated expression of both *Cxcl2* and *Il6* were sustained in the pre-exposure group throughout adipogenic differentiation. Combined these data suggest that mouse adipocytes with prior exposure to MM soluble factors produce elevated SASP proteins, which would have profound effects on tumor cells and the bone marrow microenvironment.

We next tested whether soluble factors from 5TGM1 murine myeloma cells have a similar effect on 3T3-L1 adipogenesis. Neither indirect pre-exposure to 5TGM1 cells prior to differentiation, or exposure to 5TGM1 soluble factors (conditioned media, CM) during differentiation resulted in phenotypic changes in 3T3-L1 adipocytes at terminal differentiation (**Supplementary Figure 5A**). Lipid content, as assessed *via* Oil Red-O staining and quantification, also was not significantly different (**Supplementary Figures 5B, C**). Exposure to 5TGM1 soluble factors CM (Supplementary **Figure 5D**), or with transwell indirect co-culture (**Supplementary Figure 5E**), also had no significant effects on in the secretion of over 40 cytokines, (including IL-6, CXCL1, and CXCL2), assessed *via* cytokine array; suggesting a differential effect of either myeloma cell line, or between species. To explore whether indirect co-culture with myeloma cells increases p16 or p21, commonly implicated in cellular senescence, NBM-MSCs were exposed to MM.1S and RPMI-8226 myeloma cells *via* transwell co-culture for 48 h prior to adipogenic differentiation (21 days). MSCs were harvested for total protein either immediately after co-culture, or at the end of the differentiation process. MM co-culture seemingly had very little effect on p16 or p21 in hMSCs compared to naïve (**Supplementary Figures 6A, B**), with no overall differences at the end of 21 days of adipogenic differentiation. p21 and p16 also increased in BMAT samples relative to MSCs, consistent with previous reports of PPARG stimulation of senescent markers (p16) expression (44).

In the patient dataset reanalysis (28), we compared MSCs from MM patients and from normal bone marrow (NBM), and observed significantly increased expression of 267 genes (**Supplementary Figure 7**), 36 of which encode secreted proteins (KW-0964; **Table 5**). These include key signaling molecules known to modulate the BM microenvironment including: C-C motif chemokine ligand 5 (*CCL5*), C-X-C motif chemokine ligand 8 (*CXCL8*/*IL-8*), platelet derived growth factor B (*PDGFB*), as well as transforming growth factors A and B (*TGFA*, *TGFB*). Secreted phosphoprotein 1 (*SPP1*) was also increased in MM-MSCs relative to NBM-MSCs, suggesting that myeloma MSCs may be similar to other types of cancer-associated stromal cells and adipocytes (45–47). In the DE genes reported by Todoerti et al. (40), only 21 genes were significantly upregulated in MM- *vs*. NBM-MSCs, which were predominantly enriched for factors involved in transcription, including *YY1* which may regulate p16 expression (48) (**Supplementary Figure 2**).

**Table 5:** Genes upregulated in myeloma patient-derived mesenchymal stem cells (MM-MSCs) compared to non-malignant bone marrow-MSCs (NBM-MSCs) that encode secreted proteins.

Gene Symbol	Gene Name	P-Value	Relative Expression (MM/NBM)
*ADAM23*	a disintegrin and metallopeptidase domain 23	0.00242	2.7021
*ADAMTS12*	a disintegrin-like and metallopeptidase (reprolysin type) with thrombospondin type 1 motif, 12	0.00848	2.2248
*ANGPTL4*	angiopoietin-like 4	0.03554	2.6803
*ASAH1*	N-acylsphingosine amidohydrolase 1	0.00302	2.4670
*BAGE5*	B melanoma antigen	0.00811	2.6112
*CA6*	carbonic anhydrase 6	0.00703	2.3897
***CCL5***	**chemokine (C-C motif) ligand 5**	0.00968	2.9745
*CLEC3A*	C-type lectin domain family 3, member a	0.03478	3.6175
*CSHL1*	chorionic somatomammotropin hormone like 1	0.00117	2.2147
*CST2*	cystatin SA	0.04382	2.2356
***CXCL8***	**C-X-C motif chemokine ligand 8**	0.00164	2.2690
*DPP4*	dipeptidylpeptidase 4	0.00266	2.4102
*ENPP5*	ectonucleotide pyrophosphatase/phosphodiesterase 5	0.03128	3.0575
*HPX*	hemopexin	0.03025	2.3668
*IL17D*	interleukin 17D	0.00164	2.3558
*IL1A*	interleukin 1 alpha	0.04260	3.1132
*LRRC17*	leucine rich repeat containing 17	0.01147	2.6269
*LYZ*	lysozyme	0.03481	2.5119
*MSMB*	beta-microseminoprotein	0.03234	2.1747
*NPPB*	natriuretic peptide type B	0.00038	3.9836
*OLR1*	oxidized low density lipoprotein (lectin-like) receptor 1	0.00918	3.3160
*PAPPA2*	pappalysin 2	0.04318	2.2153
***PDGFB***	**platelet derived growth factor, B polypeptide**	0.01085	2.7339
*PDGFRL*	platelet-derived growth factor receptor-like	0.04730	3.1235
*PLAT*	plasminogen activator, tissue	0.00253	2.2369
*RSPO2*	R-spondin 2	0.01459	2.3071
*SEMG1*	semenogelin 1	0.00777	3.0290
*SERPINB2*	serine (or cysteine) peptidase inhibitor, clade B, member 2	0.00028	3.7445
***SPP1***	**CXXC finger 1 (PHD domain)**	0.01160	5.0380
*SPX*	spexin hormone	0.03056	3.4733
*TFPI*	tissue factor pathway inhibitor	0.00222	2.0072
***TGFA***	**transforming growth factor alpha**	**0.03717**	2.6374
***TGFB2***	**transforming growth factor, beta 2**	**0.01007**	2.0666
*THPO*	thrombopoietin	0.00976	2.1998
*VWA5B1*	von Willebrand factor A domain containing 5B1	0.00672	4.1775
*ZG16B*	zymogen granule protein 16B	0.00238	2.4187

Reanalysis of Corre et al. 2007 Leukemia. Bold indicates discussed in text.

We further examined the expression of senescence-associated genes and observed a general trend for increased SASP gene expression (including *CXCL1*, *CXCL2*, and *IL6*) in MM-MSCs when compared to NBM-MSCs (**Figure 8E**) (28). Elevated expression of *IL6* in MM-MSCs relative to normal donor MSCs was observed in an independent experiment utilizing nanostring gene expression data (~2.5-fold, data not shown), and has been previously reported (7, 30). Overexpression of SASP genes Interleukin 1 beta (*IL1B)* and serpin family E member 1 (*SERPINE1)* were also previously reported (28). Additionally, we found that cyclin dependent kinase inhibitor 2A (*CDKN2A*) and high mobility group AT-hook 2 (*HMGA2*) were significantly increased in MM-MSCs 2- and 2.4-fold respectively; these genes encode proteins involved in senescence-associated heterochromatin foci (GO:0035985; **Supplementary Figure 7**). In the Todoerti et al. dataset, MM-MSCs overexpressed NR2F1, a transcription factor that has been linked to increased expression of *CXCL12* (49), however none of the signaling molecules listed above were included in the list of significantly different genes. In addition, among the downregulated genes were those associated with response to cytokine stimulus (GO:0071345), and the interferon signaling pathway (HSA-913531), suggesting that these processes are aberrant in MM-MSCs compared to NBM-MSCs, although the specific relationships remain unclear.

**Figure 9:**
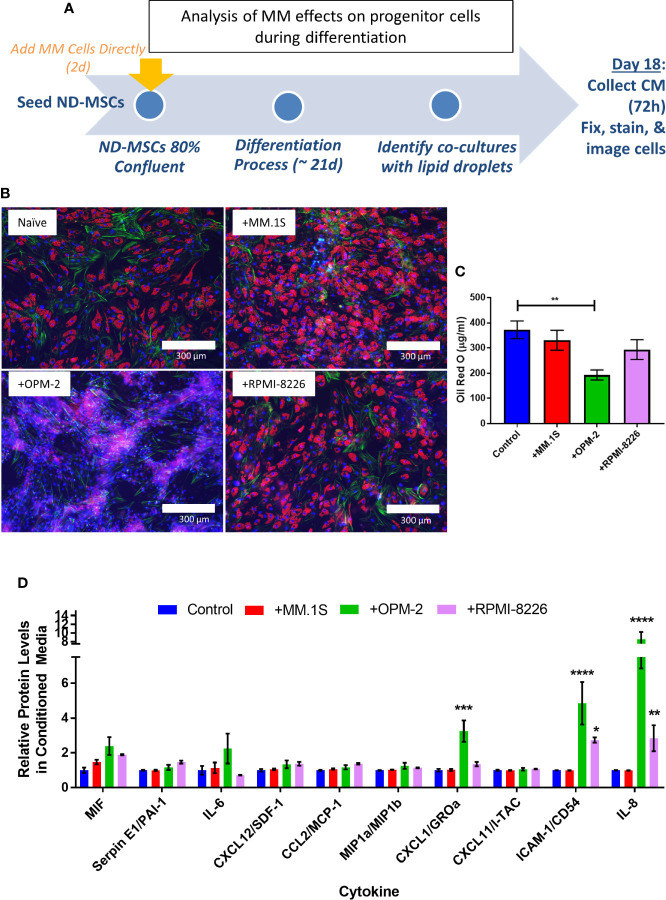
Direct co-culture of human mesenchymal stem cells (hMSCs) with myeloma cells reveals cell-line specific effects on lipid accumulation and cytokine production. Experimental design of co-culture experiment **(A)** where multiple myeloma (MM) cells (MM.1S, RPMI-8226, OPM-2) were added 2 days prior to the start of differentiation. Myeloma cells were allowed to persist during differentiation with adipogenic media. Fresh adipogenic media was incubator for 72 h prior to the collection of conditioned media. Conditioned media was collected and cells were fixed and stained on day 18 of differentiation, a few days prior to terminal differentiation. Adipocytes were fixed, stained (phalloidin=green, Oil Red-O=red, DAPI=blue), and imaged with a 10X objective after differentiation and co-culture with or without myeloma cells **(B)**; images are from one hMSC donor, but are representative of n=3 donors. Lipid content was assessed by Oil Red-O staining, elution, and quantification **(C)**. Cytokines were assessed in conditioned media by human cytokine array (R&D) **(D)**; n=3 donors for each condition (control=naïve, +MM.1S, +OPM-2, +RPMI-8226). Significance was assessed *via* two-way ANOVA. *p < 0.05, **p < 0.01, ***p < 0.001, ****p < 0.0001.

To specifically test whether MM cells can inhibit adipogenesis and promote expression of SASPs in hMSCs, we directly co-cultured NBM-MSCs with tumor cells prior to and during differentiation (**Figure 9A**). NBM-MSCs were exposed directly to MM.1S, RPMI-8226, and OPM-2 cells for 48-h prior to the initiation of differentiation. At day 0 of differentiation, the co-culture media was removed, and cells were washed gently prior to the addition of fresh adipogenic differentiation media which was changed 1–2 times weekly until day 18 at which time conditioned media was collected from the co-cultures and cells were fixed and stained for analysis (**Figure 9B**). We found that direct co-culture with MM.1S resulted in no net change in lipid content, while RPMI-8226 tumor cells a slight, non-significant decrease on lipid accumulation or content as evidenced by fluorescent microscopy and quantified by Oil Red-O elution (**Figure 9C**). However, co-culture with OPM-2 resulted in severe decreased adipogenesis as evidenced by the lack of full-fledged lipid laden adipocytes (**Figure 9B**, bottom left) and significantly reduced Oil Red-O content (**Figure 9C**). In conditioned media from MM.1S+BMAT co-cultures, we were unable to detect any differences in 36 different cytokines (**Figure 9C**). However, we observed increased inflammatory cytokines in the conditioned media of RPMI-8226 and OPM-2 co-cultures (**Figure 9C**), with significant differences in CXCL1 (OPM-2 only), ICAM-1, and IL-8 and trends for increased IL-6 and PAI-1 (*SERPINE1*).

Interestingly, we detected different responses of the myeloma cells to BMAT differentiation media (**Supplementary Figure 8A**), with severe reduction in MM.1S cell number (**Supplementary Figure 8B**), proliferation (**Supplementary Figure 8C**), coupled with high levels (~80%) of apoptosis (**Supplementary Figure 8D**). Conversely, OPM-2 cells were largely unaffected by the BMAT differentiation media, with a slight but significant reduction in cell number (**Supplementary Figure 8E**), coupled with minimal reductions in proliferation (**Supplementary Figure 8F**) and apoptosis (**Supplementary Figure 8G**). Treatment of RPMI-8226 cells with BMAT differentiation media resulted in an approximate 50% reduction in cell number (**Supplementary Figure 8H**), no effect on proliferation (**Supplementary Figure 8I**), with a moderate increase in apoptosis (**Supplementary Figure 8J**). The experimental design of this direct co-culture experiment therefor investigated the pre-exposure of MSCs (MM.1S), as well as co-culture with low (RPMI-8226), and high (OPM-2) levels of tumor cells throughout the differentiation process. This data could, in part, explain the differing levels of adipocyte differentiation and/or lipid accumulation, and its correlation with the production of SASPs, if these processes are tied to tumor cell presence, but cannot be teased apart from cell-line specific signals that might be involved in altering adipogenesis. Combined these results suggest that MM cells are likely inducing senescence in cells within the marrow niche including both adipocytes and their precursors (MSCs), which has implications for tumor cell proliferation and survival.

## Discussion

In this study, we demonstrate that exposure to myeloma cells modulates adipocyte progenitors by altering adipogenic differentiation capacity, skewing metabolism-related transcripts, and increasing the expression of inflammatory cytokines. Our data build on what is known in the field of myeloma, to demonstrate that preadipocytes that are exposed to soluble MM-derived factors are phenotypically altered. Overall, these data support the interpretation that MM cells modulate adipocytes and their precursors by inducing senescence, and stimulating production of MM-supportive cytokines and other factors that likely contribute to “the vicious cycle” (2) of bone destruction and release of growth factors fueling myeloma cells in the bone marrow.

Corre and colleagues determined that MM-MSCs exhibit extremely different expression profiles from NBM-MSCs (28). Among the upregulated genes highlighted in these findings were factors that promote lipolysis, *ANGPTL4* (50), growth differentiation factor 15 (*GDF15*) (51), and known SASPs *IL1B* and *SERPINE1*. This study also detected downregulation of chemokine C-X-C motif ligand 12 (*CXCL12, SDF1*) and insulin-like growth factor 1 (*IGF1*), and aberrant expression of genes encoding molecules involved in WNT signaling Dickkopf homolog 1 (*DKK1*) and WNT1 inducible signaling protein 1 (*WISP1*) (28). By relaxing the stringency of our reanalysis, we were able to detect additional differences in gene expression (p<0.05; FC>|2|), confirm diminished expression of osteogenic-related transcripts and growth factors, and further identify *FADS1* and *FADS2* as being elevated in MM-MSCs. The downregulation of these enzymes in MM-MSCs would likely manifest in an altered portfolio of long-chain fatty acids available to the cell, which has been tied to both osteogenic (52) and adipogenic differentiation (53). In breast cancer cells, FADS1/2 have also been linked to inflammation *via* their production of arachidonic acid (54), suggesting that their suppression in MM-MSCs may be linked to an anti-inflammatory response linked to increased cytokine signaling (55).

While we did observe some changes in transcript expression related to PPAR signaling, no significant differences were detected in *PPARG* or *CEBPA/CEBPB*, suggesting that MM-MSCs were not committed to adipogenesis, although trends for low-level elevated expression were observed in two independent datasets. Our data suggest that while these genes can be turned on in MM-MSCs during *in vitro* differentiation, traditional adipogenic differentiation is inhibited. Our findings are consistent with single cell qRT-PCR analysis of normal MSCs exposed to myeloma cells, which exhibit trends for suppression of *LEPR* and *PPARG* (26). In addition, a second dataset comparing gene expression in MM- to NBM-MSCs demonstrated significant reductions in *CEBPA*, *SOX9*, *MEIS1, ZFP36*, and *SAV1*—all of which have been linked to the promotion of adipogenic differentiation (40), supporting our hypothesis that adipogenesis is likely suppressed in MM-MSCs.

An increased number of preadipocytes within the marrow of myeloma patients has been reported, as well as supportive effects of preadipocytes and adipocytes on MM cells (8). Studies utilizing conditioned media from 3T3-L1 preadipocytes and mature adipocytes specifically highlighted a recruitment/pro-migration role for mature adipocytes, and a proliferative effect of preadipocytes on MM cells (8). These findings implicate adipocyte-lineage cells as a source of chemokines that drive myeloma bone marrow homing (8). A recent study demonstrated that BMAds from myeloma patients are reprogrammed to produce adipokines that stimulate osteoclastogenesis and suppress osteoblastogenesis (27). Our studies build on these findings by highlighting both similar and new chemokines produced by uncommitted MSCs, differentiating MSCs, and committed adipocytes, and recapitulate their production after MM exposure. Within our reanalysis of the data from Corre et al., we uncovered significantly decreased expression of *FOXA1*, a transcription factor whose suppression in cancer stem cells has been tied to increased *IL6* expression (56). We hypothesize that a similar mechanism is controlling expression of *IL6* in MM-MSCs, and potentially other SASP proteins. We specifically highlight SASP-related transcripts encoding inflammatory cytokines in each of our adipocyte-lineage experiments and demonstrate that the induction of these transcripts is sustained after differentiation. In addition, we found that direct co-culture of hMSCs with two myeloma cell lines prior to and during adipogenic differentiation resulted in increased secretion of CXCL1/GRO, IL-8, and ICAM-1 in co-culture conditioned medium samples. We also observed complete inhibition of adipogenic differentiation with OPM-2 co-culture.

Interestingly, direct co-culture with MM.1S myeloma cells, did not inhibit adipogenesis and resulted in no significant changes in secreted cytokines. A recent study by Liu et al. (57) directly co-cultured hMSCs with MM.1S cells and characterized their gene expression profiles and adipogenic differentiation capacity. After 48 h of direct co-culture, myeloma cells were removed, and single cell RNA sequencing (scRNA-seq) was used to demonstrate a shift in the gene expression profile of MSCs cultured with MM.1S cells, including enrichment of the adipokine signaling pathway. They also demonstrated impaired mineralization with osteogenic differentiation (as assessed with alizarin red staining) and elevated Oil Red O staining in MSCs previously cultured with MM.1S cells compared to naïve MSCs (57). Similar findings were also observed with ARP-1, U266, and RPMI-8,226 cells. These findings are consistent with the data presented in our current study, which utilizes a slightly different experimental design, and demonstrates no negative effect in Oil Red O staining in adipocytes directly co-cultured with either MM.1S cells or RPMI-8226 cells. Liu et al. demonstrate that MM.1S and ARP-1 cells stimulate PPARG in MSCs *via* a protein kinase C-mediated mechanism that is triggered by the binding of integrin-α4 on myeloma cells to VCAM1 on MSCs (57).

We also build on the findings of Liu et al. (57), by providing evidence that direct co-culture with OPM-2 myeloma cells eliminates or prevents adipogenic differentiation of hMSCs. The experimental design of our direct co-culture experiments differs from the study by Liu and colleagues (57), as myeloma cells were allowed to persist in culture during the adipogenic differentiation process. We observed adverse effects of the adipogenic differentiation media on the tumor cells themselves, with a severe reduction of MM.1S cells (coupled with a high level of apoptosis), a moderate reduction of RPMI-8226 cells (moderate apoptosis), and a slight reduction of OPM-2 cells (low apoptosis) after a 72-h culture. This suggests that MM.1S cells were not likely to persist throughout the adipogenic differentiation process in the co-culture system, while OPM-2 cells remained and interacted with MSCs throughout the duration of the experiment. Indeed, we observed complete inhibition of the presence of lipid laden adipocytes in OPM-2 hMSC co-cultures, and significantly elevated levels of the SASPs, GROα (*CXCL1*), IL8, and CD54 (*ICAM-1*), in the culture media. Elevated levels of IL-6 and SDF-1 (*CXCL12*) protein were also detected, although these were not significant by two-way ANOVA. Interestingly, hMSCs differentiating with RPMI-8226 in direct co-culture were able to differentiate into lipid-laden adipocytes and expressed significantly more CD54 (ICAM-1) and IL-8, and slightly more PAI-1 (SERPINE1), SDF-1, MCP-1 (CCL2), and GROα. Further investigation is required to determine whether the differences observed in direct co-culture with these three cell lines is cell-line specific, or dependent on the absence/presence of MM cells during differentiation.

Our findings are consistent with previous reports of myeloma “primed” MSCs differentiated into adipocytes such as the Mehdi et al. study that demonstrated MSC-MM co-culture for 3 days suppressed adipogenic differentiation and reduced the overall size and lipid content of those adipocytes (26). In patient samples, the authors suggest that production of small, immature IGFBP2+ adipocytes is negatively correlated with disease progression- suggesting that myeloma inhibits the formation of these cells, or that these cells are somehow utilized during MM progression (26). In 3T3-L1 adipocytes, we observed a slight, non-significant increase in *Igfbp2* expression after a two-day exposure to MM cells by indirect co-culture. The authors did report an increase in genes associated with senescence in MM patient MSCs, consistent with our findings, and adding strength to a potential role of SASPs in myeloma disease progression (31). Indeed, two key risk factors for myeloma—aging (58) and obesity (12)—have been shown to increase senescence in the bone marrow, suggesting that targeting senescent cells *via* senolytic therapies (59) may be beneficial in myeloma treatment.

We see a number of ways in which our work could translate to the clinic. First, as mentioned above, targeting senescent cells using senolytic treatment may remove senescent, myeloma-associated bone marrow adipocytes (31), or pre-adipocytes, as shown here, from the microenvironment. New ways to remove senescent cells are being developed in the field of aging, such as quercetin or dasatinib + quercetin, and the role of local senescent cells in tumor growth is becoming increasingly evident in a variety of cancers (60). The field of senotherapeutics is burgeoning and senolytics as well as senomorphics, which act to interfere with a specific senescence pathway in order to restore the appropriate cellular function, may prove useful in cancer treatment. Interestingly, dormant myeloma cells themselves (61), or MGUS (monoclonal gammopathy of undetermined) cells may also be removed through targeting senescent cells, as Weivoda et al. have recently suggested at the ASBMR, 2020 annual meeting. Others have also seen that other types of tumor cells can express senescent genes and are sensitive to senolysis (60). Overall, targeting senescence clinically may be a new means by which to target the tumor cells and the host microenvironment simultaneously.

Moreover, targeting SASPs and other factors from myeloma-associated adipocytes is another potential way to translate our findings to the bedside. For example, targeting IL6 (e.g., Johnson and Johnson’s drug sirukumab), or the IL6 receptor (e.g., Regeneron’s sarilumab) could be tailored perhaps by using BMAT biomarkers (62), knowing now that BMAds, especially myeloma-associated adipocyte lineage cells, are a source of IL6. Better alignment with patient populations for enhanced precision medicine could also be considered based on our data, for therapies targeting the CXCL12/CXCR4 axis, such as the CXCR4 inhibitor AMD3100 (plerixafor) (63), or the CXCL12 antagonist NOX-A12 (64). Our data may also help explain the ability of treatment with plerixafor to overcome bortezomib resistance and mobilize stem cells and immune cells, which was recently reported in a phase I/II trial (65), by providing new insight into the players in the bone marrow niche involved in this pathway. Interestingly, new data *in vitro* has shown that SDF-1α stimulation of CXCR4 on MM cells may up-regulate the expression of IL-6 through the activation of the PI3K/AKT, suggesting that the IL6 and CXCR4/CXCL12 pathways overlap in MM cells (64). Overall, our study provides insight into the mechanism of action of drugs targeting the CXCL12/CXCR4 axis, the IL6/IL6R axis, or other SASP protein signaling pathways in MM, and suggests that targeting proteins identified herein may lead to new therapeutic avenues.

In conclusion, our studies demonstrate that adipocyte-lineage cells are dramatically altered by MM cells. MSCs exposed to myeloma-derived soluble factors exhibited reduced differentiation capacity, and elevated expression of senescence-related transcripts including MM-supportive *Il6/IL6*. Mouse preadipocytes exposed to MM.1S myeloma cells can differentiate, but accumulate less lipid and exhibit aberrant gene expression, including upregulation of key SASP genes; however this was not observed with exposure to 5TGM1 murine myeloma soluble factors. Direct co-culture of hMSCs with human myeloma cell lines revealed extreme differences in the effects of myeloma cells on adipogenic differentiation, with an increase in adipogenesis observed in response to MM.1S co-culture, and an inhibitory response observed in response to OPM-2 co-culture, which stimulated SASP production. The induction of SASP gene expression by MM cells in adipocyte lineage cells underlines the importance of future studies to examine whether SASPs promote MM tumor initiation, disease stage transition, or resistance to traditional chemotherapies. Moreover, the myeloma-derived factors that induce senescence and modulate adipogenesis and in pre-adipocytes should be explored in future experiments. Our studies indicate that myeloma cells induce senescence in adipocyte-lineage cells and add to the building knowledgebase that SASP proteins are involved in MM pathogenesis.

## Data Availability Statement

The datasets presented in this study can be found in online repositories. The names of the repository and accession number can be found here: Gene Expression Omnibus (GEO)(GSE143267).

## Ethics Statement

The animal study was reviewed and approved by The Maine Medical Center Research Institute (Scarborough, ME, USA) Institutional Animal Care and Use Committee (IACUC).

## Author Contributions

HF, CF, and MR conceptualized and designed the study. HF, CF, and MR developed the methodology. HF, CF, and SC acquired the data. HF, CF, SC, HD, and MR analyzed and interpreted the data. HF and MR wrote, reviewed, and/or revised the manuscript. All authors contributed to the review and revision of the manuscript. HF, HD, AD, and MR provided administrative support. HF, SC, and CF provided technical support. HF and MR supervised the study. All authors contributed to the article and approved the submitted version.

## Funding

Funding for this work was supplied by the NIH’s National Institute of General Medical Sciences (NIH P30 GM106391, P30GM103392, P20GM121301, and U54GM115516) from their Molecular Phenotyping and Professional Development Cores, and from the NIH/NCI R37CA245330. The authors’ work is also supported by start-up funds from the Maine Medical Center Research Institute, a pilot from the MGH Center for Skeletal Research (NIH/NIAMS P30 AR066261), and the American Cancer Society (RSG-19-037-01-LIB and #IRG-16-191-33; Reagan PI). The content is solely the responsibility of the authors and does not necessarily represent the official views of the National Institutes of Health.

## Conflict of Interest

The authors declare that the research was conducted in the absence of any commercial or financial relationships that could be construed as a potential conflict of interest.
