# Supplementary material for: Multiple Myeloma Cells Alter Adipogenesis, Increase Senescence-Related and Inflammatory Gene Transcript Expression, and Alter Metabolism in Preadipocytes
Source: Front Oncol. 2021 Feb 18;10:584683. doi: 10.3389/fonc.2020.584683 (PMC7930573; doi:10.3389/fonc.2020.584683)
Supplement: Supplementary file 1 [file DataSheet_1.docx]

Supplementary Material

## Supplementary Figures

**
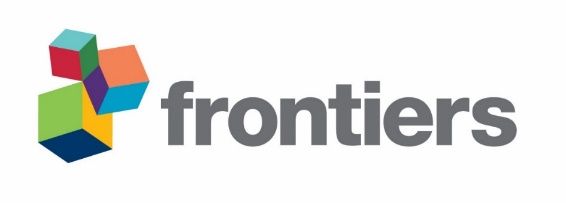
**

**
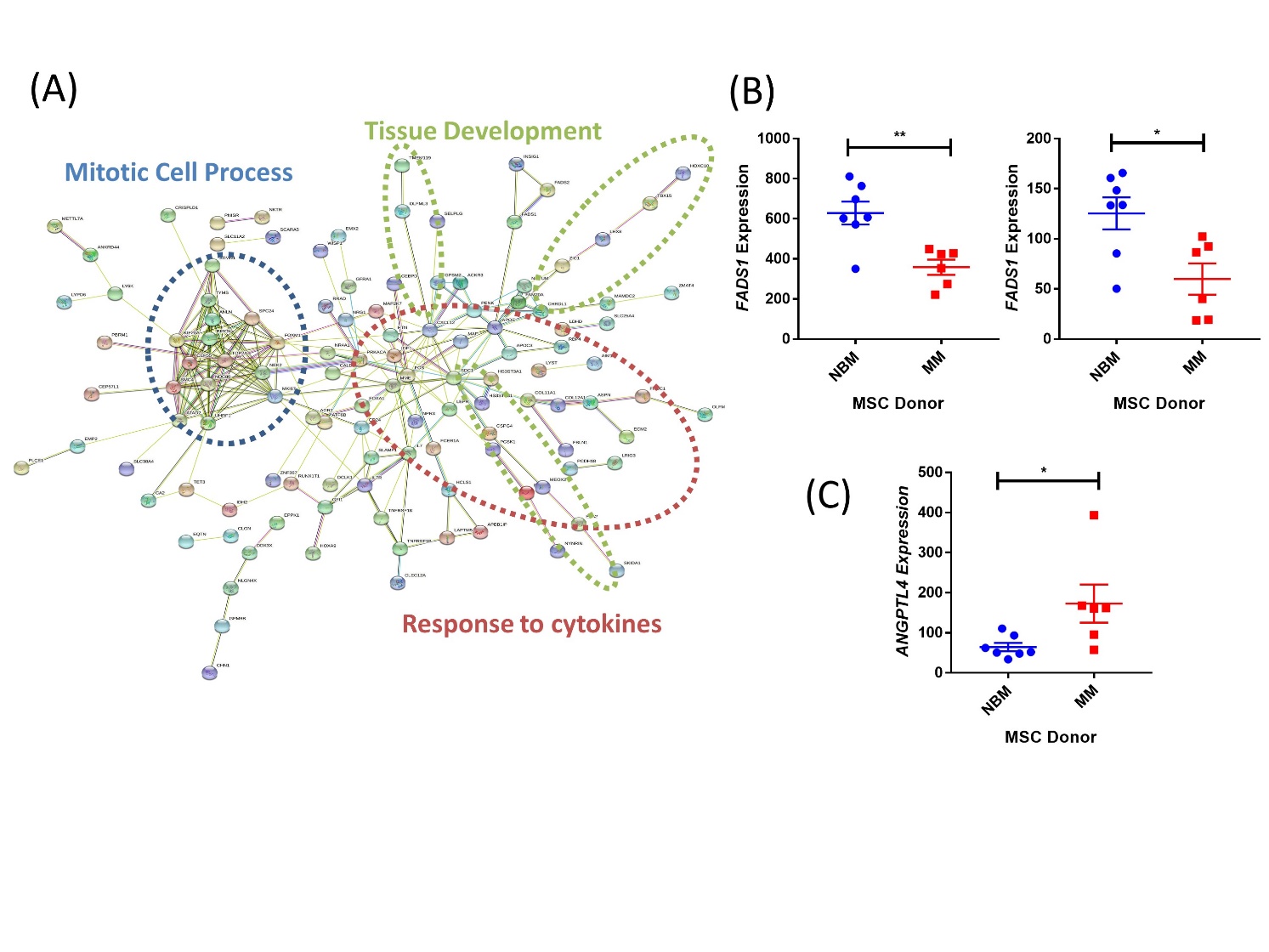
**

**Supplemental Figure 1. Expression of critical genes altered in myeloma patient MSCs.** String-db analysis of significantly downregulated (p<0.05, FC≤-2) transcripts in MM-MSCs compared to NBM-MSCs (A). Additional transcript expression for *FADS1* (B) and *ANGPTL4* (C). Analysis of publicly available data from Corre et al. 2007, *Leukemia*.


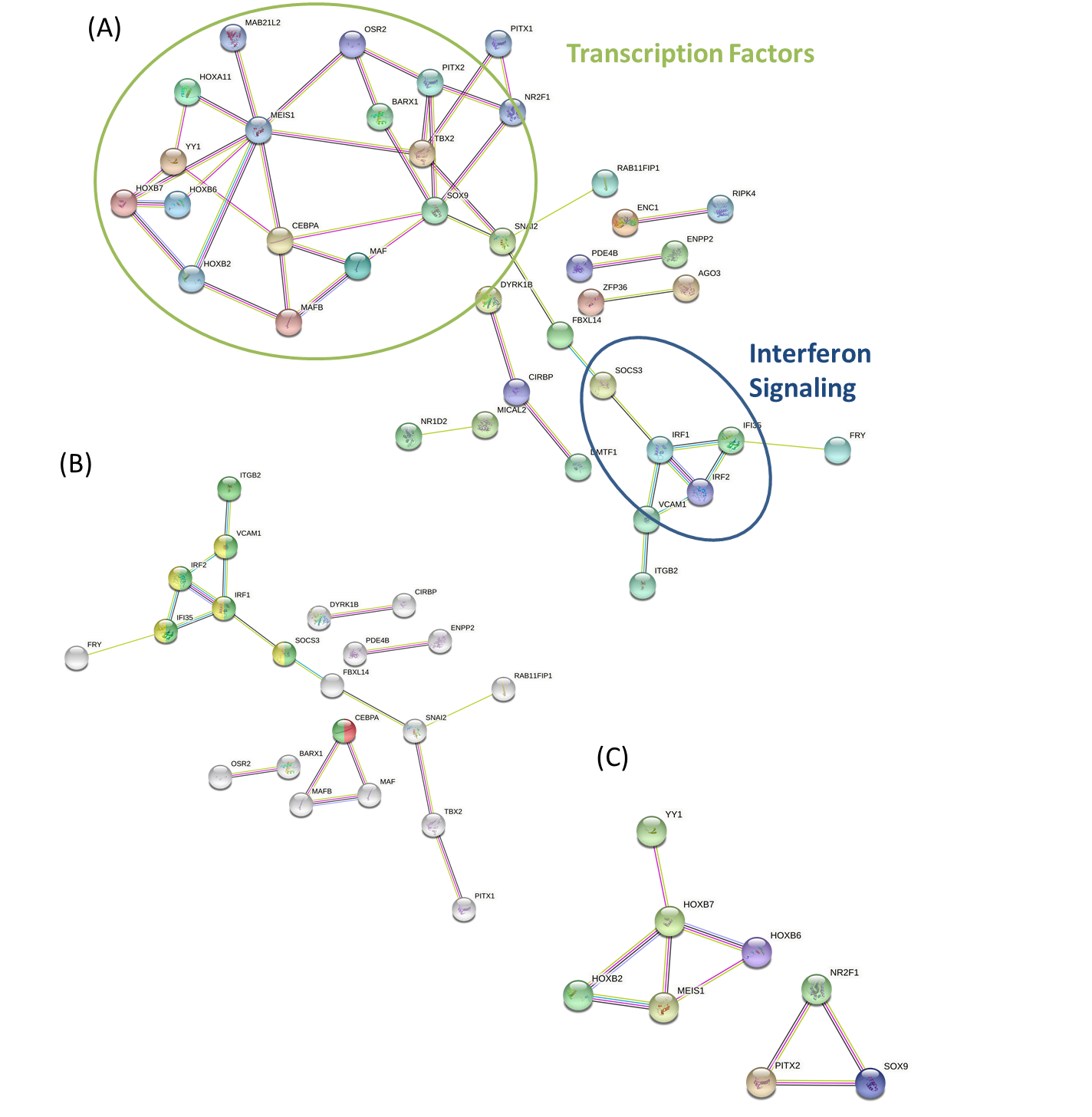


**Supplementary Figure 2.** **Differentially expressed genes in myeloma patient MSCs.** String-db analysis of all differentially expressed genes (A), as well as downregulated (B) and upregulated genes in MM-MSCs compared to NBM-MSCs. Nodes highlighted in downregulated genes: red= positive regulation of fat cell differentiation (GO: 0045600), green= cellular response to cytokine stimulus (GO:0071345), yellow=interferon signaling (HSA-9133531). Analysis of publicly available data from Todoerti et al. 2007, *Experimental Hematology*.


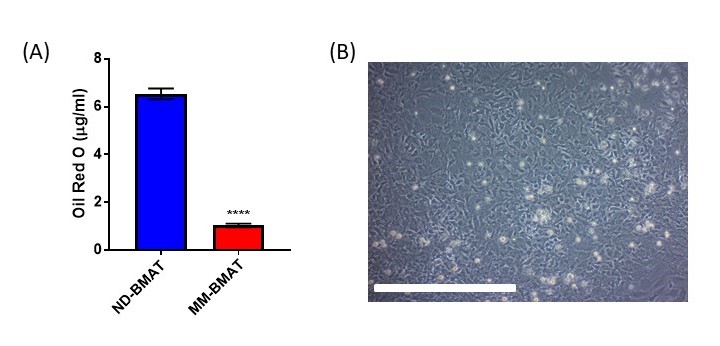


**Supplementary Figure 3. Adipogenic capacity in myeloma-associated stromal cells.** Quantification of oil red o (A) from ND-MSCs differentiated into bone marrow adipose tissue (ND-BMAT) and MM-MSCs differentiated into bone marrow adipose tissue (MM-BMAT) using 21 days of adipogenic media (n=16 wells, 1 donor). Undifferentiated mouse MSCs (B), imaged with 10X objective; scale bar = 500 µm.

**
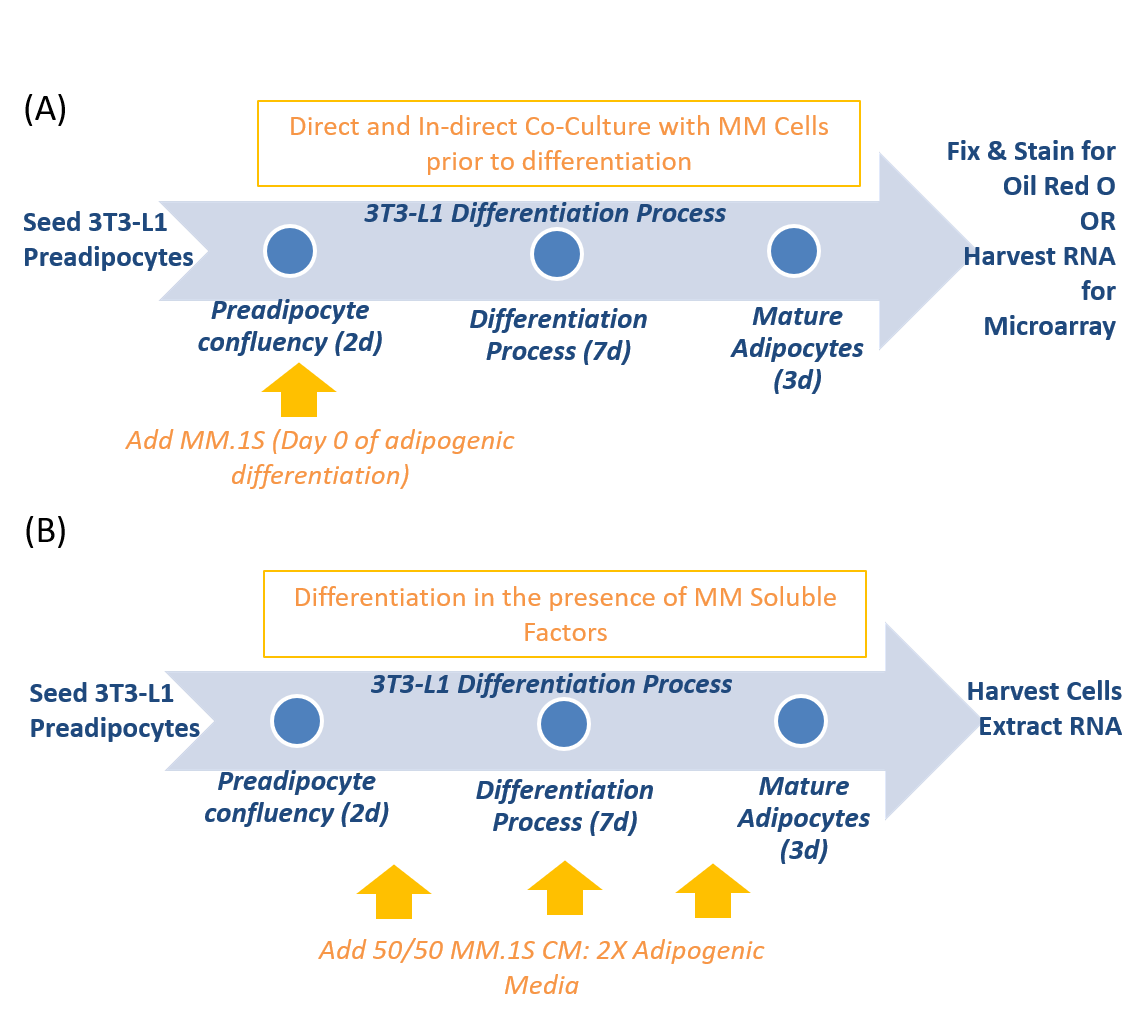
**

**Supplemental Figure 4. Adipogenic differentiation of preadipocytes is inhibited by MM cells.** Experimental design of experiments comparing 3T3-L1 preadipocytes exposed to MM cells for 48 hours prior to differentiation (A). Experimental design of qPCR experiment comparing the differentiation of 3T3-L1 preadipocytes in the presence or absence of MM soluble factors by exposure to myeloma conditioned media (CM) (B).

**Supplemental Figure 5. Characterization of the effects of 5TGM1 soluble factors on 3T3L1 adipogenesis**. 3T3-L1 adipocytes were differentiated after exposure to 5TGM1 cells via transwell (TW) for 48 hours, or in the presence 5TGM1 conditioned media (CM) for 7 days prior to the collection of conditioned media and fixation and staining with Oil Red O (A; scale bar= 300 µm). Lipid content was assessed by oil red-o elution and quantification (B-C). 3T3-L1 adipocytes exhibit no significant differences in cytokine production in response to either (D) 5TGM1 conditioned media administered during differentiation or (E) indirect co-culture of 5TGM1 cells via transwell co-culture prior to differentiation; n=3 experiments. Conditioned media was collected for 48 hours at the end of adipogenic differentiation and assessed via mouse cytokine array.


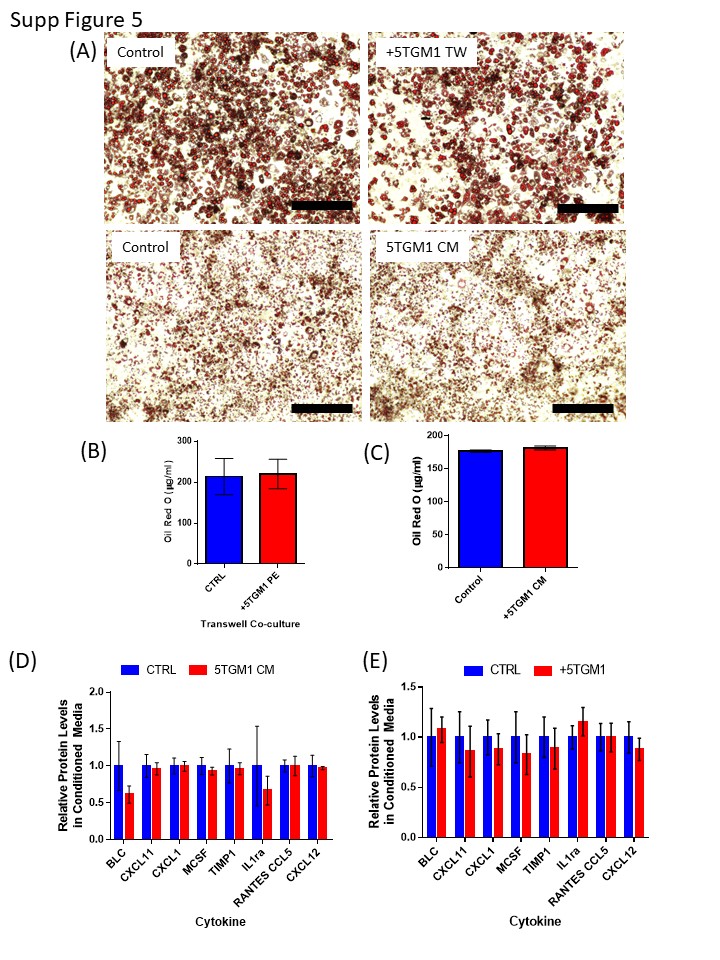

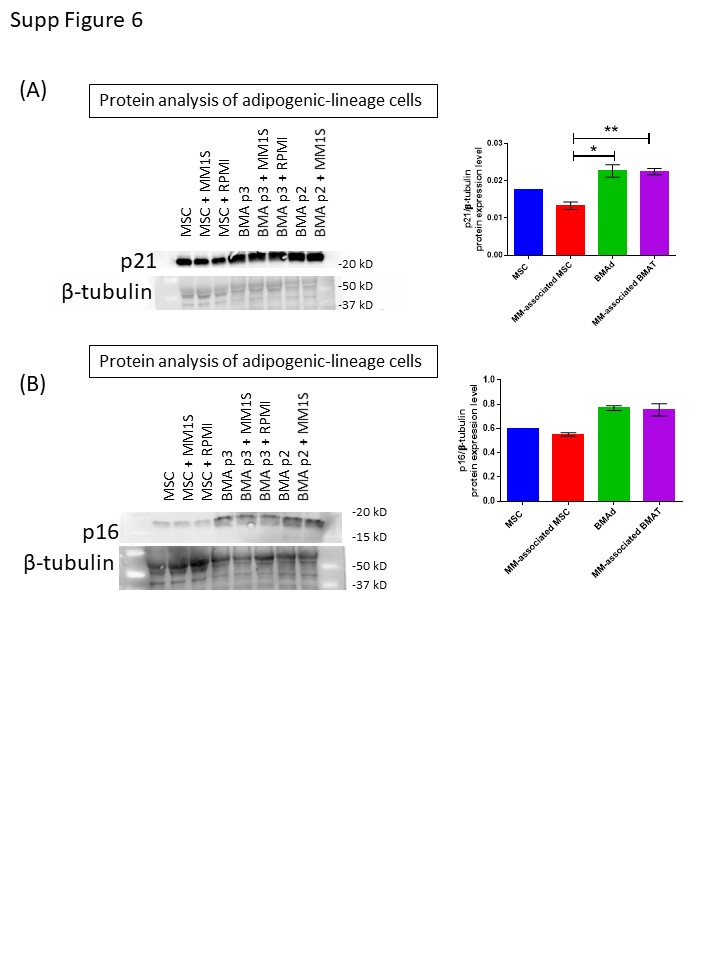


**Supplemental Figure 6. Effects of soluble myeloma-derived factors on senescence-associated markers.** p21(A) and p16 (B) protein levels were assessed in hMSCs exposed to MM.1S cells via transwell, immediately after exposure (MM-associated MSC), or after 21 days of differentiation following pre-exposure with MM.1S cells (MM-associated BMAT). Two-way ANOVA with Tukey’s post-hoc analysis was used to assess significance.

**
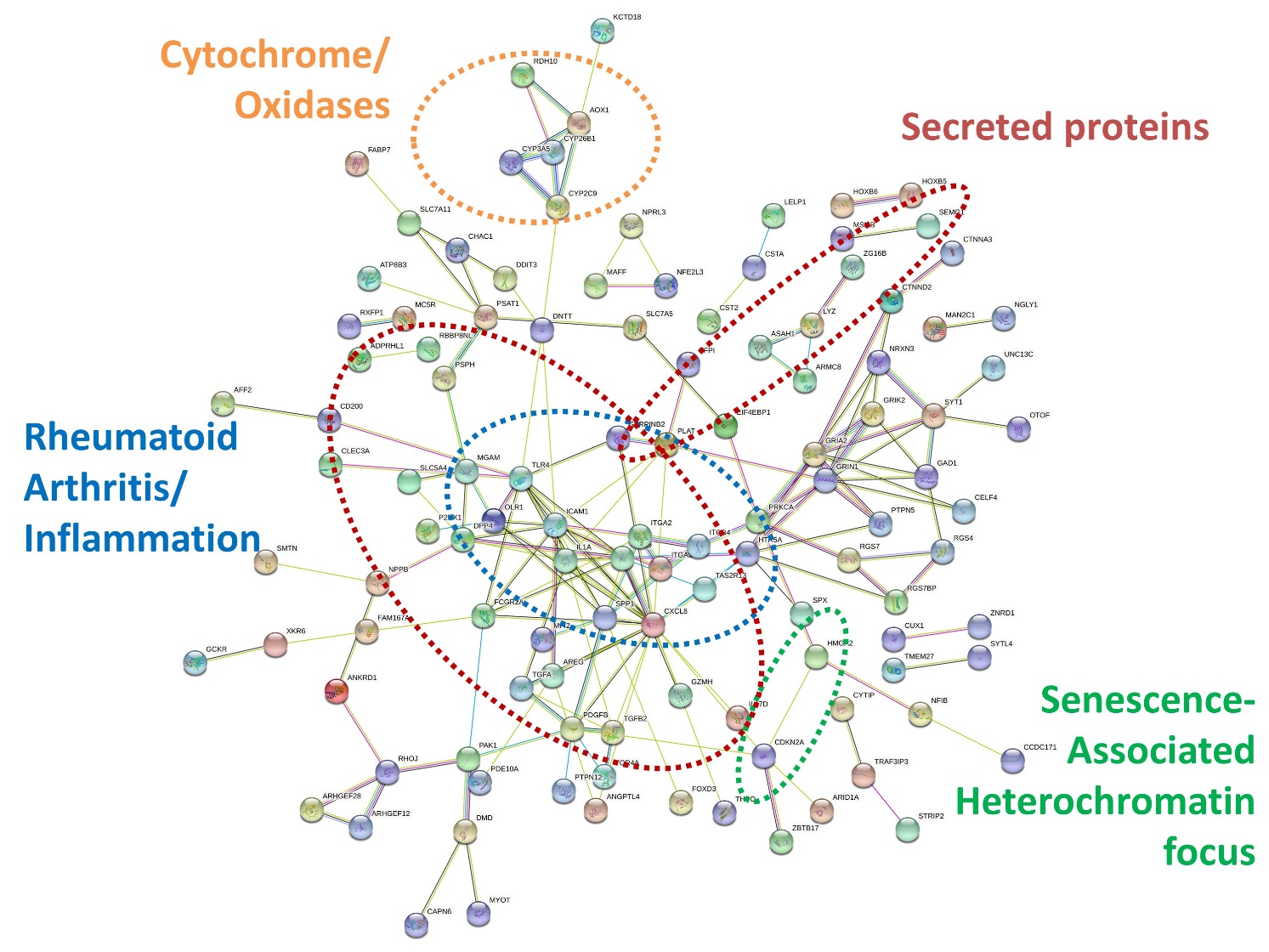
**

**Supplemental Figure 7. Upregulated transcripts in MM-MSCs reveal increased expression of inflammatory cytokines.** String-db analysis of significantly upregulated (p<0.05, FC≥2) transcripts in MM-MSCs compared to NBM-MSCs. Analysis of publicly available data from Corre et al. 2007, *Leukemia*.

**
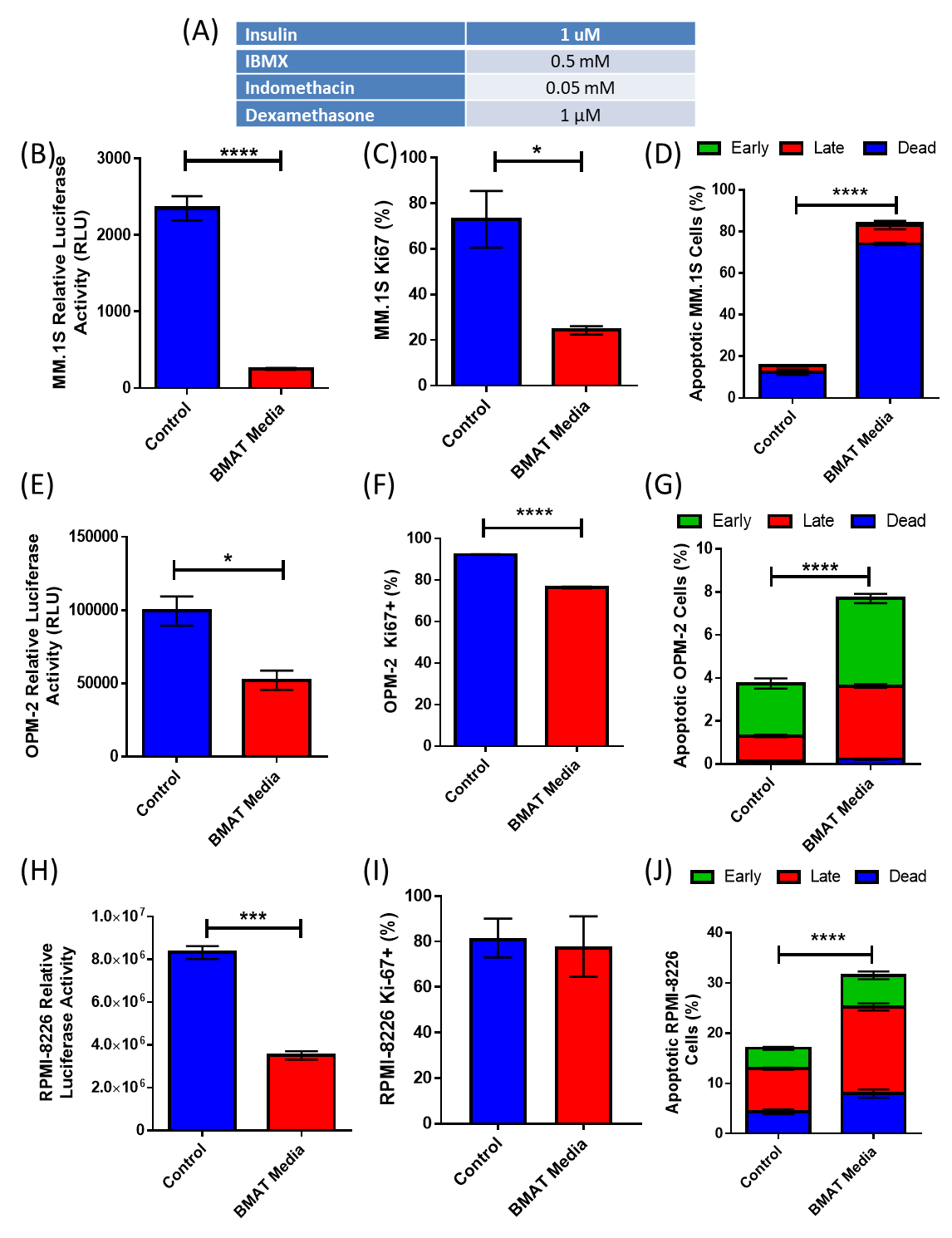
**

**Supplemental Figure 8. Myeloma cell lines are affected by adipogenic differentiation media with implications for co-culture experiments.** Concentrations of various components used in adipogenic differentiation (AD) media (A). Effects of AD on MM.1S^gfp+luc+^ (B-D) cell number (B), proliferation (C), and apoptosis (D); OPM-2^mcherry+luc+^ (E-G) cell number (E), proliferation (F), and apoptosis (G); RPMI-8226 (H-J) cell number (H), proliferation (I), and apoptosis (J). Cell number was assessed by luciferase activity by luciferin spike in for MM.1S and OPM-2, and CellTiter Glo in RPMI-8226. Proliferation and apoptosis were assessed utilizing flow cytometery with Ki67 labeling, and Annexin V/DAPI labeling respectively. *p < 0.05, ***p < 0.001, ****p < 0.0001.

## Supplementary Tables

**Supplementary Table 1.** **Significantly upregulated genes in 3T3-L1 adipocytes exposed to MM.1S cells prior to adipogenic differentiation.** 105 genes were upregulated by a fold-change of >1.5 and met the p-value cutoff of p<0.05.

| **Gene Symbol** | **RefSeq** | **p-value** | **Fold-Change (MM1S vs. Control)** |
| --- | --- | --- | --- |
| *Camp* | NM_009921 | 0.0469083 | 3.72546 |
| *Irak3* | NM_028679 | 0.0111517 | 3.64475 |
| *C4a* | NM_011413 | 0.00494647 | 2.40538 |
| *Lcn2* | NM_008491 | 0.0128835 | 2.34621 |
| *C4b* | NM_009780 | 0.000832202 | 2.30039 |
| *Fgl1* | NM_145594 | 0.0190904 | 2.24146 |
| *Id3* | NM_008321 | 0.00221037 | 2.1954 |
| *A4galt* | NM_001004150 | 0.0477032 | 2.18086 |
| *Hp* | NM_017370 | 0.00487906 | 2.17003 |
| *Steap4* | NM_054098 | 0.0147021 | 2.12538 |
| *Clu* | NM_013492 | 0.00801329 | 2.12235 |
| *Itih4* | NM_001159299 | 0.0151637 | 2.10771 |
| *Ereg* | NM_007950 | 0.0358901 | 2.08879 |
| *Grem1* | NM_011824 | 0.000907029 | 2.02669 |
| *St14* | NM_011176 | 0.00341382 | 2.02231 |
| *Ccl20* | NM_001159738 | 0.0445496 | 1.99698 |
| *Pde8b* | NM_001170669 | 0.0364435 | 1.97279 |
| *Il6* | NM_031168 | 0.0479097 | 1.95355 |
| *Frmpd1* | NM_001081172 | 0.0127849 | 1.92557 |
| *Sall4* | NM_175303 | 0.0214699 | 1.91396 |
| *Dram1* | NM_027878 | 0.00235566 | 1.90215 |
| *Ins2* | NM_001185083 | 0.0389931 | 1.88911 |
| *Hivep3* | NM_010657 | 0.000224723 | 1.88139 |
| *Trpv5* | NM_001007572 | 0.0444293 | 1.86192 |
| *Rgs16* | NM_011267 | 0.0299276 | 1.85746 |
| *Defb34* | NM_183035 | 0.00397214 | 1.83704 |
| *Gm10717* | ENSMUST00000075573 | 0.0189944 | 1.83146 |
| *Wdr64* | NM_029453 | 0.0289294 | 1.82466 |
| *Cyp2c50* | NM_001167875 | 0.0441895 | 1.81041 |
| *Id1* | NM_010495 | 0.00067358 | 1.78665 |
| *Cd14* | NM_009841 | 0.0270081 | 1.77449 |
| *Slc9a9* | NM_177909 | 0.0126696 | 1.76749 |
| *Ostn* | NM_198112 | 0.0341075 | 1.76103 |
| *Pde1b* | NM_001285890 | 0.0336929 | 1.76035 |
| *Olfr142* | NM_146984 | 0.02391 | 1.75737 |
| *Gabra2* | NM_008066 | 0.0475318 | 1.75475 |
| *Lrrn4cl* | NM_001013019 | 0.0105767 | 1.74632 |
| *Ap1g2* | NM_007455 | 0.0222297 | 1.73513 |
| *Olfr498* | NM_146307 | 0.0433498 | 1.72864 |
| *Olfr325* | NM_207153 | 0.0213064 | 1.72192 |
| *Vstm2a* | NM_145967 | 0.0398428 | 1.72105 |
| *Abca8a* | NM_153145 | 0.0259487 | 1.70572 |
| *Dusp15* | NM_001159376 | 0.0393336 | 1.7053 |
| *Myh13* | NM_001081250 | 0.00282801 | 1.69065 |
| *Ccl6* | NM_009139 | 0.0189107 | 1.6839 |
| *Wdr66* | BC138176 | 0.00588127 | 1.6835 |
| *Tomm40l* | NM_001037170 | 0.0230187 | 1.68227 |
| *Tmem69* | NM_177670 | 0.00955769 | 1.6818 |
| *Bpifb9a* | NM_175167 | 0.0392147 | 1.68152 |
| *Mrgprb13* | XM_884524.1 | 0.00937781 | 1.67676 |
| *Lgi2* | NM_144945 | 0.0368476 | 1.67356 |
| *1700040L02Rik* | NM_028491 | 0.0236681 | 1.67328 |
| *Gm11168* | ENSMUST00000177722 | 0.00993062 | 1.6704 |
| *Frrs1l* | NM_001142965 | 0.0375884 | 1.65743 |
| *Tex15* | NM_031374 | 0.00820703 | 1.64894 |
| *Atf3* | NM_007498 | 0.0428791 | 1.64782 |
| *Olfr522* | NM_146952 | 0.0123422 | 1.63793 |
| *Sla2* | NM_029983 | 0.000381624 | 1.63517 |
| *1110008P14Rik* | NM_198001 | 0.047498 | 1.63482 |
| *Mageb16-ps1* | NR_033647 | 0.038158 | 1.61754 |
| *Mettl13* | NM_144877 | 0.0159868 | 1.61356 |
| *Gm10800* | ENSMUST00000099683 | 0.000188034 | 1.60875 |
| *Lgals2* | NM_025622 | 0.00662435 | 1.60721 |
| *Iqcf5* | NM_029300 | 0.0170504 | 1.60694 |
| *Gm10720* | ENSMUST00000099050 | 0.0479136 | 1.60378 |
| *Slpi* | NM_011414 | 0.0270927 | 1.60228 |
| *Rab3b* | NM_023537 | 0.0432969 | 1.60045 |
| *Hspa1l* | NM_013558 | 0.0492045 | 1.59856 |
| *Wfdc21* | NM_183249 | 0.0391166 | 1.59771 |
| *Cyp2f2* | NM_007817 | 0.00374465 | 1.59765 |
| *Slc38a6* | NM_001037717 | 0.0483509 | 1.59723 |
| *P2rx3* | NM_145526 | 0.00445631 | 1.59327 |
| *Krt16* | NM_008470 | 0.00755071 | 1.59229 |
| *Lilrb4a* | NM_013532 | 0.00777996 | 1.59162 |
| *Six4* | NM_011382 | 0.0134765 | 1.5911 |
| *Il1a* | NM_010554 | 0.00179691 | 1.58414 |
| *Cxcl12* | NM_001012477 | 0.0124545 | 1.57787 |
| *Sp7* | NM_130458 | 0.0099214 | 1.57681 |
| *Camkk2* | NM_001199676 | 0.00226949 | 1.57502 |
| *Msx3* | NM_010836 | 0.00906309 | 1.57104 |
| *Gm10721* | ENSMUST00000143083 | 0.0318448 | 1.56975 |
| *Ackr3* | NM_001271607 | 0.0136647 | 1.56614 |
| *Trnp1* | NM_001081156 | 0.0342377 | 1.56432 |
| *Nkx1-1* | ENSMUST00000173348 | 0.0162189 | 1.56218 |
| *4930597O21Rik* | ENSMUST00000065878 | 0.00646505 | 1.56002 |
| *Necab2* | NM_054095 | 0.0193049 | 1.5541 |
| *Mup4* | NM_008648 | 0.0232165 | 1.54728 |
| *Clca3a1* | NM_009899 | 0.0251766 | 1.54198 |
| *Defb10* | NM_139225 | 0.0331585 | 1.54177 |
| *Abcc2* | NM_013806 | 0.00969951 | 1.53815 |
| *Defb22* | NM_001002791 | 0.0428185 | 1.53665 |
| *Clca3a2* | NM_030601 | 0.0460015 | 1.53313 |
| *Gm10718* | ENSMUST00000099046 | 0.0306704 | 1.53249 |
| *Parvg* | NM_001162500 | 0.0438121 | 1.52903 |
| *Abcg4* | NM_138955 | 0.000856541 | 1.52894 |
| *Clvs1* | NM_028940 | 0.0138639 | 1.51896 |
| *Gm7714* | NM_001110779 | 0.00433872 | 1.51384 |
| *Adh6-ps1* | NR_033581 | 0.0390807 | 1.51333 |
| *Gm5460* | NM_001034880 | 0.0448829 | 1.51325 |
| *Klhl13* | NM_026167 | 0.049952 | 1.51289 |
| *H2-Q1* | NM_010390 | 0.0497745 | 1.50653 |
| *Gm13084* | NM_001005371 | 0.0494844 | 1.50646 |
| *Ttr* | NM_013697 | 0.00351779 | 1.50367 |
| *Gm10676* | ENSMUST00000098778 | 0.0398489 | 1.5026 |

**Supplementary Table 2:** **Significantly downregulated genes in 3T3-L1 adipocytes exposed to MM.1S cells prior to adipogenic differentiation.** 179 genes were downregulated by a fold-change of <-1.5 and met the p-value cutoff of p<0.05.

| **Gene Symbol** | **RefSeq** | **p-value** | **Fold-Change (MM1S vs. Control)** |
| --- | --- | --- | --- |
| *Brca2* | NM_001081001 | 0.0171 | -1.5035 |
| *Fhl2* | NM_001289533 | 0.0324068 | -1.50374 |
| *Fam63b* | NM_172772 | 0.0125922 | -1.50737 |
| *Angel2* | NM_001199020 | 0.0470518 | -1.50768 |
| *Gm10197* | ENSMUST00000086887 | 0.0320131 | -1.50796 |
| *Col11a1* | NM_007729 | 0.0414051 | -1.51222 |
| *Dhrs1* | NM_026819 | 0.0284668 | -1.51224 |
| *Plk4* | NM_011495 | 0.0139595 | -1.51402 |
| *Fbn1* | NM_007993 | 0.018272 | -1.51429 |
| *Poc1b* | NM_027740 | 0.00966812 | -1.51662 |
| *Mvd* | NM_138656 | 0.0171304 | -1.51722 |
| *Ppp1r3b* | NM_177741 | 0.0340856 | -1.52038 |
| *Gabre* | NM_017369 | 0.0366721 | -1.52063 |
| *Zfp956* | NM_178898 | 0.0224934 | -1.52224 |
| *Cldn15* | NM_021719 | 0.0130111 | -1.52241 |
| *1810024B03Rik* | NM_198630 | 0.0151062 | -1.52545 |
| *Rhox4b* | NM_021300 | 0.00945153 | -1.52623 |
| *Rhox4d* | NM_001039695 | 0.00945153 | -1.52623 |
| *Mdc1* | NM_001010833 | 0.0071191 | -1.52665 |
| *Zfp677* | NM_172486 | 0.0151232 | -1.52748 |
| *Tubgcp5* | NM_146190 | 0.0468047 | -1.52845 |
| *Tada3* | NM_133932 | 0.000742718 | -1.52966 |
| *Dna2* | NM_177372 | 0.0485433 | -1.53004 |
| *Atp10a* | NM_009728 | 0.0279789 | -1.53105 |
| *Spon2* | NM_133903 | 0.0103539 | -1.53162 |
| *Tgfbi* | NM_009369 | 0.0349827 | -1.53488 |
| *Prl2c5* | NM_181852 | 0.012584 | -1.5364 |
| *Gpsm2* | NM_029522 | 0.0477398 | -1.53677 |
| *Ercc6l2* | NM_001013608 | 0.0449196 | -1.53717 |
| *Fbln5* | NM_011812 | 0.0107608 | -1.53839 |
| *Ltbp1* | NM_019919 | 0.0161087 | -1.54154 |
| *Slc16a3* | NM_001038653 | 0.0310034 | -1.54213 |
| *Hhat* | NM_144881 | 0.00578237 | -1.54228 |
| *Ppih* | NM_001110129 | 0.0300988 | -1.54281 |
| *Dcaf4* | NM_001165256 | 0.0374682 | -1.54374 |
| *Hmgn5* | NM_016710 | 0.0249982 | -1.54472 |
| *Pbx3* | NM_016768 | 0.00290204 | -1.54485 |
| *Zfp787* | NM_001013012 | 0.0128167 | -1.546 |
| *Slc9a3r1* | NM_012030 | 0.0426109 | -1.54909 |
| *Zfp101* | NM_009542 | 0.0167394 | -1.55026 |
| *Ehbp1* | NM_001252515 | 0.00788485 | -1.55079 |
| *Pfkp* | NM_019703 | 0.0177103 | -1.55418 |
| *Fgf10* | NM_008002 | 0.0177603 | -1.55648 |
| *Thrsp* | NM_009381 | 0.0183445 | -1.56041 |
| *Sdpr* | NM_138741 | 0.0427777 | -1.56555 |
| *Mid1* | NM_010797 | 0.0188915 | -1.56972 |
| *Tbc1d15* | NM_025706 | 0.0470392 | -1.57399 |
| *Cep295* | NM_176976 | 0.0183626 | -1.57409 |
| *Ankrd37* | NM_001039562 | 0.0116051 | -1.57426 |
| *Zfp354c* | NM_013922 | 0.0368258 | -1.57777 |
| *Gm5595* | NM_001008427 | 0.0176765 | -1.57809 |
| *Cep152* | NM_001081091 | 0.0264192 | -1.57946 |
| *Heph* | NM_001159627 | 0.00747809 | -1.58055 |
| *Nid2* | NM_008695 | 0.00273867 | -1.58228 |
| *Ppox* | NM_008911 | 0.0170211 | -1.58288 |
| *Shcbp1* | NM_011369 | 0.0175554 | -1.58393 |
| *9430069I07Rik* | NM_001256161 | 0.0107426 | -1.58435 |
| *Cox6a2* | NM_009943 | 0.00523889 | -1.5847 |
| *Zfp389* | NR_026798 | 0.0109225 | -1.58505 |
| *Pdlim2* | NM_001253736 | 0.0108775 | -1.58814 |
| *Tmco4* | NM_029857 | 0.0105541 | -1.58871 |
| *Lig1* | NM_001083188 | 0.0217257 | -1.59277 |
| *Gucd1* | NM_175133 | 0.0258265 | -1.59368 |
| *Cpt1a* | NM_013495 | 0.0159351 | -1.6035 |
| *Ephb3* | NM_010143 | 0.0387207 | -1.60354 |
| *Homer1* | NM_001284189 | 0.0304224 | -1.60356 |
| *Kcnd3* | NM_001039347 | 0.046505 | -1.60479 |
| *Mxi1* | NM_001008542 | 0.0321953 | -1.60773 |
| *Ide* | NM_031156 | 0.049538 | -1.60932 |
| *Epha7* | NM_001122889 | 0.045558 | -1.6137 |
| *Hirip3* | NM_172746 | 0.00142482 | -1.6204 |
| *Arhgef28* | NM_012026 | 0.0150965 | -1.62109 |
| *Gpld1* | NM_008156 | 0.0430644 | -1.6225 |
| *Clec3b* | NM_011606 | 0.0158569 | -1.62357 |
| *Pdk4* | NM_013743 | 0.00233555 | -1.63411 |
| *Osr1* | NM_011859 | 0.00270567 | -1.63422 |
| *Pir* | NM_027153 | 0.0246715 | -1.64381 |
| *Mospd2* | NM_029730 | 0.00936462 | -1.64973 |
| *Fam57b* | NM_001146347 | 0.0107732 | -1.6551 |
| *Plekhh2* | NM_177606 | 0.00655194 | -1.65745 |
| *Kif23* | NM_024245 | 0.026251 | -1.6586 |
| *Gfpt2* | NM_013529 | 0.0450597 | -1.6606 |
| *Pdgfra* | NM_001083316 | 0.0248934 | -1.66454 |
| *Cxxc1* | NM_028868 | 0.0163446 | -1.66455 |
| *Map3k6* | NM_016693 | 0.000885982 | -1.66539 |
| *Grk5* | NM_018869 | 0.0162979 | -1.66921 |
| *Flrt2* | NM_201518 | 0.043774 | -1.67171 |
| *Insl6* | NM_013754 | 0.0434193 | -1.67629 |
| *Jup* | NM_010593 | 0.012693 | -1.67685 |
| *Uggt2* | NM_001081252 | 0.000505067 | -1.67777 |
| *Maged2* | NM_001199246 | 0.0106697 | -1.67949 |
| *Slc1a4* | NM_018861 | 0.0492975 | -1.68038 |
| *Pcyt1b* | NM_177546 | 0.014312 | -1.68108 |
| *Fzd2* | NM_020510 | 0.00463055 | -1.68259 |
| *Cyp2ab1* | NM_183158 | 0.0192478 | -1.6834 |
| *Ddx26b* | NM_172779 | 0.0268944 | -1.69777 |
| *Stard4* | NM_133774 | 0.0368311 | -1.70004 |
| *Mmp9* | NM_013599 | 0.0171499 | -1.70377 |
| *Ccl11* | NM_011330 | 0.0265249 | -1.7045 |
| *Errfi1* | NM_133753 | 0.0121621 | -1.70497 |
| *Cemip* | NM_030728 | 0.037487 | -1.70602 |
| *Hoxd3os1* | NR_027899 | 0.00656545 | -1.70783 |
| *Nt5e* | NM_011851 | 0.0117729 | -1.70982 |
| *Clmn* | NM_001040682 | 0.0131005 | -1.7119 |
| *Rdh10* | NM_133832 | 0.0442222 | -1.71248 |
| *Nsdhl* | NM_010941 | 0.0151529 | -1.71566 |
| *N4bp2* | NM_001024917 | 0.0319872 | -1.71615 |
| *Medag* | NM_027519 | 0.00510369 | -1.7206 |
| *Tfap2a* | NM_001122948 | 0.00991587 | -1.72635 |
| *Fasn* | NM_007988 | 0.0364073 | -1.72769 |
| *Mad2l1* | NM_019499 | 0.0268562 | -1.73104 |
| *Rcbtb1* | NM_027764 | 0.0393083 | -1.73566 |
| *Sc5d* | NM_172769 | 0.00861774 | -1.74396 |
| *Scd2* | NM_009128 | 0.00722872 | -1.74797 |
| *Iffo2* | NM_001205173 | 0.039912 | -1.75264 |
| *Ang3* | NM_001123394 | 0.00354553 | -1.76201 |
| *Lmln* | NM_172823 | 0.0311676 | -1.76787 |
| *Ccdc82* | NM_025534 | 0.00631421 | -1.77364 |
| *Msi1* | NM_008629 | 0.00204097 | -1.78508 |
| *Apaf1* | NM_001042558 | 0.000406525 | -1.79901 |
| *Foxo4* | NM_018789 | 0.00950658 | -1.80286 |
| *Gcnt4* | NM_001166065 | 0.012132 | -1.80355 |
| *Postn* | NM_001198765 | 0.0185354 | -1.80465 |
| *Tmem80* | NM_001141950 | 0.0258898 | -1.80969 |
| *Plekha8* | NM_001001335 | 0.00902589 | -1.81507 |
| *Aspn* | NM_001172481 | 0.0372649 | -1.822 |
| *B130024G19Rik* | NR_045850 | 0.0423879 | -1.83619 |
| *Mrgprf* | NM_145379 | 0.00304001 | -1.83984 |
| *Rad18* | NM_001167730 | 0.0481986 | -1.84148 |
| *Srpx2* | NM_001083895 | 0.00424439 | -1.85576 |
| *Cetn4* | NM_145825 | 0.0282721 | -1.86177 |
| *Slc40a1* | NM_016917 | 0.0136254 | -1.88526 |
| *Cdo1* | NM_033037 | 0.00705248 | -1.90253 |
| *Rap2a* | NM_029519 | 0.00853954 | -1.93238 |
| *Hspa4l* | NM_011020 | 0.01532 | -1.93257 |
| *Olfm3* | NM_001286750 | 0.00419473 | -1.9478 |
| *Acly* | NM_001199296 | 0.00982447 | -1.94818 |
| *Srebf2* | NM_033218 | 0.00774787 | -1.96953 |
| *Pmvk* | NM_026784 | 0.000952557 | -1.97263 |
| *Mss51* | NM_029104 | 0.0212877 | -1.97768 |
| *Akr1b7* | NM_009731 | 0.0268748 | -1.98328 |
| *Fads1* | NM_146094 | 0.0086805 | -1.98531 |
| *Cenpe* | NM_173762 | 0.00208714 | -1.99684 |
| *Ffar4* | NM_181748 | 0.0301225 | -2.04529 |
| *Igfbp5* | NM_010518 | 0.00878757 | -2.06053 |
| *Stx1b* | NM_024414 | 0.0433419 | -2.06237 |
| *Fdft1* | NM_010191 | 0.00127213 | -2.08306 |
| *Prdm9* | NM_144809 | 0.0297217 | -2.08673 |
| *Ecscr* | NM_001033141 | 0.00421446 | -2.08679 |
| *Fads2* | NM_019699 | 0.0022095 | -2.10536 |
| *Msmo1* | NM_025436 | 0.0161765 | -2.1191 |
| *Mfap4* | NM_029568 | 0.0327087 | -2.13687 |
| *S1pr1* | NM_007901 | 0.0258692 | -2.15617 |
| *Slc9a3r2* | NM_023055 | 0.00410087 | -2.163 |
| *Krt80* | NM_028770 | 0.0246504 | -2.22153 |
| *Cdhr1* | NM_130878 | 0.00971536 | -2.23092 |
| *Ttc21b* | NM_001047604 | 0.0291247 | -2.24825 |
| *Dhcr24* | NM_053272 | 0.00360391 | -2.26937 |
| *Efnb2* | NM_010111 | 0.00756599 | -2.30152 |
| *Msln* | NM_018857 | 0.0118966 | -2.43582 |
| *Fdps* | NM_001253751 | 0.00842156 | -2.4588 |
| *Ctla2a* | NM_001145799 | 0.0129969 | -2.51163 |
| *Lrrc17* | NM_028977 | 0.0415032 | -2.54021 |
| *Fmod* | NM_021355 | 0.0113768 | -2.54232 |
| *Fbxo47* | NM_001081435 | 0.0429175 | -2.55708 |
| *Sqle* | NM_009270 | 0.00224033 | -2.61451 |
| *3110039M20Rik* | NR_026733 | 0.0161059 | -2.73556 |
| *Prg4* | NM_001110146 | 0.00208021 | -2.73926 |
| *Ogn* | NM_008760 | 0.010968 | -2.80385 |
| *Insig1* | NM_153526 | 0.0181098 | -2.86322 |
| *Itgbl1* | NM_145467 | 0.000345048 | -2.89127 |
| *Igf2* | NM_001122736 | 0.000665603 | -2.95494 |
| *Cyp51* | NM_020010 | 0.0051486 | -3.08925 |
| *Osr2* | NM_054049 | 0.00280817 | -3.19102 |
| *Utp14b* | NM_001001981 | 0.0254226 | -3.24598 |
| *Sema3a* | NM_001243072 | 0.00142074 | -3.28218 |
| *Apod* | NM_007470 | 0.0290703 | -3.74711 |
| *Il1rl1* | NM_001025602 | 0.00946742 | -4.05537 |
| *Aldoc* | NM_009657 | 0.0124467 | -4.18555 |
| *Agtr2* | NM_007429 | 0.00203888 | -9.0694 |
